# Boosting POM‐Ionosolv Biorefining of Lignocellulosic Biomass by Using Redox‐Balanced Polyoxometalate Catalysts in Methanolic Ionic Liquid Reaction Media

**DOI:** 10.1002/cssc.202501034

**Published:** 2025-09-10

**Authors:** Stefanie Wesinger, Aleksandra Rabiner, Suhaib Nisar, Leonhard Schill, Mariusz Grzegorz Kubus, Maximilian J. Poller, Anders Riisager, Agnieszka Brandt‐Talbot, Jason P. Hallett, Jakob Albert

**Affiliations:** ^1^ Institute of Technical and Macromolecular Chemistry University of Hamburg Bundesstraße 45 20146 Hamburg Germany; ^2^ Department of Chemical Engineering Imperial College London Bone Building, South Kensington Campus London SW7 2AZ UK; ^3^ Department of Chemistry Imperial College London Molecular Sciences Research Hub White City Campus, 82 Wood Lane W12 0BZ UK; ^4^ Department of Chemistry Technical University of Denmark Kemitorvet, 207 2800 Kgs. Lyngby Denmark

**Keywords:** cellulose pulp, green chemistry, lignocellulosic biomass, methyl formate, polyoxometalate, polyoxometalate‐ionosolv biorefining

## Abstract

This article presents an advanced iteration of the polyoxometalate (POM)‐Ionosolv concept to generate biobased methyl formate in high yield and a bleached cellulose pulp from lignocellulosic biomass in a single‐step operation by using redox‐balanced POM catalysts and molecular oxygen in alcoholic ionic liquid (IL) mixtures. The performance of the three Ionosolv‐ILs triethylammonium hydrogen sulfate ([TEA][HSO_4_]), *N*,*N*‐dimethylbutylammonium hydrogen sulfate ([DMBA][HSO4]), and tributylmethylphosphonium methyl sulfate ([TBMP][MeSO_4_]), mixed with methanol (MeOH) (30/70 wt%), is evaluated by methyl formate yield from extracted hemicellulose and lignin as well as purity of the bleached cellulose pulp in the presence of various Keggin‐type POMs. The redox‐balanced H_8_PVMnMo_10_O_40_ POM catalyst in [TBMP][MeSO_4_]/MeOH emerge as the most effective combination, achieving 20% methyl formate yield from commercial beech wood. The glucan content in the bleached cellulose‐enriched solid consisted is over 90%, demonstrating that the use of MeOH drastically improved lignin extraction in parallel with full hemicellulose extraction. The cellulose is highly susceptible to enzymatic hydrolysis, generating a pure and concentrated cellulosic glucose stream. The formed solid catalyst complex is examined in detail to reveal its chemical nature as POM‐IL‐complex. The approach is applicable to disparate types of lignocellulosic biomass, including hardwood, softwood, and grass.

## Graphical abstract

This study presents an advanced iteration of the polyoxometalate (POM)‐Ionosolv concept to generate biobased methyl formate in high yield and a bleached cellulose pulp from lignocellulosic biomass in a single‐step operation by using redox‐balanced POM catalysts and molecular oxygen in alcoholic ionic liquid mixtures.

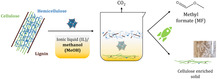

## 1 Introduction

Considering the acceleration of global warming in recent years, it is imperative for the chemical industry to transition to the utilization of renewable resources. A particularly promising renewable resource is lignocellulosic biomass, chiefly comprised of cellulose, hemicellulose, and lignin.^[^ ^1^ ^]^ Cellulose is a polysaccharide consisting of *β*‐D‐glucopyranose units linked together by *β*‐1,4‐glycosidic bonds, constituting 23–50 wt% of lignocellulosic biomass. Cellulose has gained significant interest as an environmentally friendly and renewable feedstock for the synthesis of biobased materials, biofuels, and chemicals.^[^ ^1^, ^2^, ^3^ ^]^ Its abundance, high mechanical strength, biodegradability, and renewability makes it attractive in a variety of applications, including the packaging, paper and textiles, bioplastics, and scaffolds for biomedical products and other advanced materials.^[^ ^1^, ^2^, ^3^ ^]^ Hemicellulose is a complex branched group of polysaccharides, consisting of heterogeneous mixtures of pentoses (xylose, arabinose) and hexoses (mannose, glucose, galactose) as well as acids such as acetic, galacturonic, and glucuronic acid. *β*‐1,4‐glycosidic, and occasionally, *β*‐1,3‐glycosidic bonds link the subunits. Hemicellulose constitutes 20–40 wt% of lignocellulosic biomass. The hemicellulose in hardwoods contains mostly xylans and some glucomannans, while softwood mainly contains glucomannans and some xylans, arabinogalactans, xyloglucans, and glucans.^[^ ^3^, ^4^, ^5^ ^]^ The third major biopolymer in lignocellulosic biomass is lignin. It consists of phenylpropanoid units with one or two main aromatic subunits, guaiacyl and syringyl, and a range of aliphatic linkages, such as β‐O‐4 ether, β‐β (resinol), and β‐5 (phenylcoumaran). Lignin has a random structure, nonhydrolysable linkages, is hydrophobic, and self‐reactive (lignin condensation), which protects against microbial invasion and obstructs lignin extraction.^[^ ^6^, ^7^, ^8^, ^9^ ^]^ Every component in lignocellulosic biomass can be valorized into products used for various applications, including biofuels, biochemicals, biopolymers, and pharmaceuticals.^[^ ^10^, ^11^, ^12^ ^]^

The occurrence of multiple components necessitates fractionative pretreatment of lignocellulosic biomass, which is carried out in the presence of solvents, such as water, organic solvents (organosolv), or organic salts, including ionic liquids (ILs) and deep eutectic solvents.^[^ ^13^ ^]^ Among the various fractionation approaches, the use of low‐cost IL water mixtures is a promising avenue (ionosolv fractionation) due to the low volatility of the ILs, which facilitates solvent recovery.^[^ ^14^ ^]^ ILs are salts that exist as liquids typically below 100 °C. They consist of large, asymmetric carbon‐containing cations and small, typically nonorganic anions.^[^ ^13^, ^15^ ^]^ Some examples of ILs used successfully in lignocellulosic biomass fractionation are low‐cost salts, such as triethylammonium hydrogen sulfate [TEA][HSO_4_] and *N,N*‐dimethylbutylammonium hydrogen sulfate [DMBA][HSO_4_].^[^ ^14^, ^16^ ^]^ Another IL known for effectively dissolving lignin is tributylmethylphosphonium methyl sulfate, [P_1444_][MeSO_4_] or [TBMP][MeSO_4_].^[^ ^13^ ^]^

Previous studies have demonstrated the efficacy of combining ionosolv fractionation and in situ oxidation utilizing a polyoxometalate (POM) catalyst and molecular oxygen.^[^ ^17^, ^18^, ^19^ ^]^ POMs are anionic polynuclear metal‐oxo clusters that exhibit a large structural diversity and tunable properties. Several studies have investigated the valorization of biomass using POM catalysts to produce value‐added chemicals, such as formic acid, lactic acid, and levulinic acid.^[^ ^20^, ^21^, ^22^, ^23^ ^]^ The combination of [TEA][HSO_4_] and water (30:70 wt%) enabled extraction of hemicellulose and lignin from various biomass, which were oxidized in situ to formic acid in the presence of the HPA‐5 (H_8_PV_5_Mo_7_O_40_) POM catalyst and molecular oxygen, while generating a cellulose‐enriched solid with a high glucan content as a second product. So far, the purity of the cellulose‐enriched solid has been a limiting factor for further utilization in biotechnology, which could be due to the low solubility of lignin in water.^[^ ^19^, ^24^ ^]^

The combination of ethanol as cosolvent with [TEA][HSO_4_] for ionosolv‐organosolv pretreatment was found beneficial in a previous study,^[^ ^19^ ^]^ while MeOH has also demonstrated to be a superior extraction solvent for lignin from lignocellulosic biomass in several studies.^[^ ^25^, ^26^ ^]^ MeOH has also been investigated as a suitable solvent for POM‐catalyzed biomass oxidation, showing that methyl formate as a promising building block in C_1_ chemistry^[^ ^27^ ^]^ was obtained as the only oxidation product at exceptional >99% selectivity,^[^ ^28^ ^]^ resulting from manipulation of the HPA‐5 catalyst by forming a stable vanadate‐methanol‐complex in solution completely suppressing undesired CO_2_ formation.^[^ ^29^ ^]^ Hence, the combination of MeOH with IL as solvent for combined lignocellulose fractionation and in situ POM‐catalyzed oxidation has the potential for a step‐change in biorefining of lignocellulosic biomass. **Table** **1** underlines the importance of the different combined systems in this study.

**Table 1:** Importance of different systems combined in this study.

	Methanol	ILs	POM catalyst
Function	Organosolv‐fractionation	Ionosolv‐fractionation	Catalytic oxidation
Advantages	• Increasing lignin fractionation[25, 26] • Suppressing undesired CO_2_ formation in combination with POM catalyst[28, 29]	• Effective fractionation of complex biomass[13] • Low volatility—enhancing the reaction safety[14] • High quality of cellulosic residue after fractionation[13, 14, 16]	• Effective in in‐situ oxidation of biomass in different solvents, like water, methanol or aqueous ILs[17, 18, 19, 20, 21, 22, 23] • Valorization of the liquid fraction of the combined fractionation method without a second step

The present study investigates the potential of this advanced POM‐Ionosolv concept applying an efficient POM catalyst in an IL/MeOH reaction mixture for the production of a highly cellulose‐enriched solid and methyl formate as a liquid product with high yield and selectivity in a single‐step process. The use of several ILs displaying high hemicellulose and lignin extraction was examined in combination with methanol as a co‐solvent and redox‐balanced POM catalysts to avoid over‐oxidation of the dissolved fraction to CO_2_. The interaction between the IL and the POM catalyst was subjected to rigorous investigation, and the purification and separation of the solid product were demonstrated. Moreover, a diverse range of lignocellulosic biomass, including softwood, hardwood, and grass, were evaluated as potential substrates.

## 2 Results and Discussion

### 2.1 Selection of a Suitable Solvent for the Enhanced POM‐Ionosolv Concept

A limitation of the biomass fractionation via the original POM‐Ionosolv concept, as demonstrated by Bukowski et al.^[^ ^17^, ^18^ ^]^ and Wesinger et al.,^[^ ^19^ ^]^ is the low extraction efficiency of lignin into the employed [TEA][HSO_4_] with water as a cosolvent. Conversely, methanol has been suggested as a highly effective solvent for dissolving lignin.^[^ ^29^, ^30^ ^]^ Therefore, an improved alcoholic POM‐Ionosolv 2.0 concept was developed, combining the ionosolv fractionation method and the organosolv fractionation method using methanol. To the best of our knowledge, the combination with a POM catalyst and this novel fractionation method was not applied before.

Three different ILs were selected and investigated for their ability to enhance hemicellulose and lignin extraction: [TEA][HSO_4_] as a benchmark from previous studies,^[^ ^17^, ^18^ ^]^ [DMBA][HSO_4_],^[^ ^31^ ^]^ and [TBMP][MeSO_4_].^[^ ^32^ ^]^ The first two ILs are well‐known, low‐cost IL options established for ionosolv fractionation, with [DMBA][HSO_4_] typically demonstrating improved lignin extraction relative to [TEA][HSO_4_].^[^ ^31^ ^]^ The third IL was recognized for its effective lignin extraction^[^ ^16^ ^]^ but is not as cost‐effective as the other two ILs. However, phosphonium ILs are known for their high stability against oxidizing agents.^[^ ^32^ ^]^ All ILs were mixed with MeOH in a ratio of 30 wt% IL and 70 wt% MeOH, based on the optimal IL*‐*cosolvent ratio determined by Bukowski et al.^[^ ^18^ ^]^ For clarity, the solvent mixtures are named as follows: [TEA][HSO_4_]/MeOH (30 wt%/70 wt%) is referred to as methanolic TEA solvent, [DMBA][HSO_4_]/MeOH (30 wt%/70 wt%) as methanolic DMBA solvent, and [TBMP][MeSO_4_]/MeOH (30 wt%/70 wt%) as methanolic TBMP solvent. Beech wood was chosen as the substrate to ensure better comparability with previous studies on the POM‐Ionosolv concept.^[^ ^17^, ^18^, ^19^ ^]^

**Figure 1:**
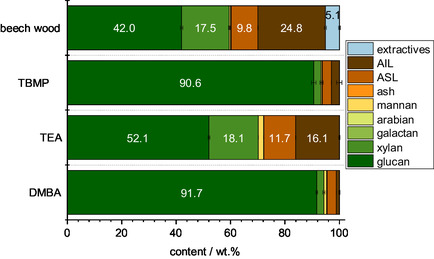
Compositional analysis of pure beech wood and the processed solids using different IL/MeOH solvents via NREL/TP‐510‐42618.^[^ ^47^ ^]^ Reaction conditions for preparing the solids: 120 °C, 24 h, 1000 rpm, 30 bar oxygen at reaction temperature, 0.5 g beech wood, and 10 g solvent (either methanolic TEA solvent, methanolic DMBA solvent, or methanolic TBMP solvent). ASL = acid soluble lignin, AIL = acid insoluble lignin.

After fractionating biomass in the three solvents without oxidative catalyst, the solids’ composition, morphology, and elemental composition were determined (**Figure** **1**). The compositional analysis of the solids and the untreated beech wood indicates that glucan could be enriched with all three solvents. Notably, the methanolic TEA solvent performed worse than the other two solvents, with 52.1% glucan in the solid compared to 42.0% glucan in the raw beech wood, showing only limited cellulose enrichment. In contrast, the other two solvents extracted lignin and hemicellulose near‐quantitatively, with the glucan content in the DMBA and TBMP treated solid exceeding 90% (91.7% and 90.6%, respectively).

**Figure 2:**
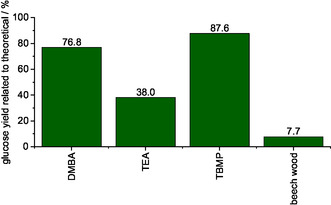
Enzymatic saccharification yields of pure beech wood in comparison to cellulose‐enriched solid processed using different IL/MeOH solvents analyzed via LAP NREL/TP‐5100‐63351.^[^ ^51^ ^]^ Reaction conditions producing solids: 120 °C, 24 h, 1000 rpm, 30 bar of oxygen at reaction temperature, 10 g solvent (either methanolic TEA solvent, methanolic DMBA solvent, or methanolic TBMP solvent), 0.5 g beech wood.

This high glucan content also correlated with the morphology of the solids (see Figure S1, Supporting Information). While the solid processed with methanolic TEA solvent had an appearance similar to unprocessed beech wood, the solids processed with methanolic DMBA and methanolic TBMP solvents appeared white and defibrillated, similar to cotton balls. The appearance suggests that combining the IL with MeOH as a fractionation medium in the presence of oxygen generated a bleached cellulose pulp consisting of over 90 wt% glucan. It is possible that the cellulose content is underestimated, which is a function of the laboratory analytical procedure (LAP) compositional analysis protocol, which was developed for raw lignocellulosic biomass. To investigate the accessibility of this cellulose‐enriched solid to cellulase enzymes, an enzymatic saccharification according to LAP NREL/TP‐5100‐63351^[^ ^51^ ^]^ was performed. **Figure** **2** shows that all processed solids generated a higher glucose yield than the unprocessed beech wood. The methanolic TEA solid generated the lowest glucose yield (38%), consistent with its lower glucan content (52.1 wt%) compared to the other IL/MeOH solvents tested. Conversely, the highest glucose yield of 87.6% was achieved with methanolic TBMP, while the methanolic DMBA solvent generated a glucose yield of 76.8%.

### 2.2 Combining Suitable Solvents with the HPA‐5 Catalyst

**Figure 3:**
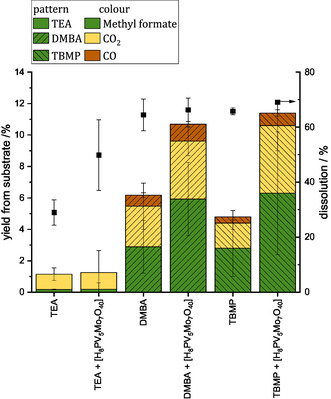
Product yields using different IL/MeOH solvents with the reference HPA‐5 catalyst. Reaction conditions: 120 °C, 24 h, 1000 rpm, 30 bar oxygen at reaction temperature, 10 g solvent (either methanolic TEA solvent, methanolic DMBA solvent, or methanolic TBMP solvent), 0.5 g beech wood, 0.18 g catalyst HPA‐5 (1 mmol g^−1^ catalyst to solvent).

To valorize the extracted hemicellulose and lignin, the HPA‐5 POM catalyst was employed, as several studies have shown that this compound is highly promising for the in situ oxidation of lignin and hemicellulose in various solvents.^[^ ^17^, ^18^, ^20^, ^22^, ^33^ ^]^ All three methanolic IL solvents were screened with HPA‐5 to oxidize the dissolved hemicellulose and lignin into the C_1_ building block methyl formate. The comparison of the results obtained with and without catalyst for the different solvents is shown in **Figure** **3**. The dissolution of the carbon fraction from beech wood was determined via the carbon content of the substrate determined by carbon, hydrogen, nitrogen, sulfur (CHNS) elemental analysis and the solid phase product and is shown on the right, while the methyl formate yield is shown on the left. Detailed calculations can be found in the Experimental Section.

Dissolution of carbon can be used to evaluate the fractionation performance while methyl formate yield is used to quantify the effectiveness of oxidative conversion of the dissolved compounds. Figure 3 shows that the methanolic TEA solvent extracted about 30% of the carbon fraction in the original biomass. Adding the HPA‐5 catalyst enhanced the dissolution to about 50%, likely due to the additional Brønsted acidity provided by the POM catalyst, which facilitates the cleavage of carbon–carbon (C—C) bonds in the lignocellulosic biomass. In general, the other two solvents showed a higher dissolution whereby the enhancement was less pronounced. The difference in the dissolution of the carbon fraction with and without HPA‐5 catalyst in the methanolic DMBA solvent was 1.9% (64.4% without catalyst and 66.3% with catalyst), within a standard deviation of 5%. For the methanolic TBMP solvent, the difference was 4.3% (above the standard deviation of 1.5%) with 65.8% dissolution without catalyst and 69.1% with catalyst.

The yield of methyl formate increased in the presence of the HPA‐5 catalyst in both the methanolic DMBA solvent (from 2.9% to 5.9%) and the methanolic TBMP solvent (from 2.9% to 6.1%). However, this was not the case for the methanolic TEA solvent, as it showed only a negligible methyl formate yield. The formation of the undesired byproduct CO_2_ also increased with the presence of HPA‐5 catalyst in both the methanolic DMBA solvent (from 2.6% to 3.7%) and the methanolic TBMP solvent (from 1.4 to 3.2%).

The stability of the IL/MeOH solvents under reaction conditions with HPA‐5 present was further investigated in the absence of substrate. As evident in the ^13^C nuclear magnetic resonance (NMR) spectra of the reaction mixtures (Figure S2–S4, Supporting Information), all the IL/MeOH solvents formed traces of methyl formate resulting from acid‐catalyzed MeOH oxidation, as previously shown by Tatibouët et al. using a vanadium‐incorporated POM catalyst (H_3+n_PV_v_Mo_12‐n_O_40_ on K_3_PMo_12_O_40_).^[^ ^34^ ^]^ Consequently, the methyl formate yields were corrected accordingly as described in the Experimental Section. In the tested methanolic IL solvents, acidic sites were present in the IL as well as the POM catalyst, and the latter also contributed redox‐active sites. The termination of the reaction depends on both acidity and redox activity, hence, it is assumed that the partial oxidation of MeOH to methyl formate was facilitated by the high redox activity of HPA‐5, due to the incorporation of five vanadium atoms into the POM structure, in combination with the acidic ILs. Therefore, balancing both redox‐active and Brønsted acid sites within the POM structure is required. The acidity of the solvent media play also an important role for the overall reaction media stability.

**Figure 4:**
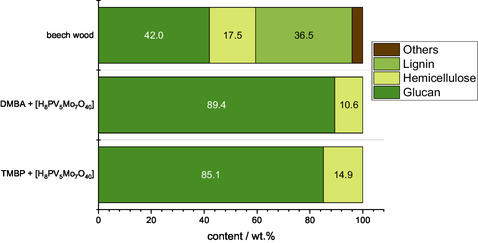
Compositional analysis of pure beech wood in comparison to the processed cellulose‐rich solid analyzed by NIR spectroscopy after reaction with the HPA‐5 catalyst. Reaction conditions: 120 °C, 24 h, 1000 rpm, 30 bar oxygen at reaction temperature, 10 g solvent (either methanolic TEA, methanolic DMBA, or methanolic TBMP), 0.5 g beech wood, 0.18 g catalyst HPA‐5 (1 mmol g^−1^ catalyst to solvent).

As the methanolic TEA solvent generally showed lower performance in terms of hemicellulose and lignin dissolution and methyl formate yield, only the composition of the processed solids formed in the DMBA and TBMP solvents with HPA‐5 catalyst were analyzed by near infrared (NIR) spectroscopy.^[^ ^35^ ^]^ As shown in **Figure** **4**, the catalyst influenced the composition of the processed solids, resulting in a slightly lower glucan content with the catalyst than without it (see Figure 1) (methanolic DMBA solvent: 2.3% lower, methanolic TBMP solvent: 5.5% lower). This could be due to catalyst precipitation (elaborated in more detail in the next section), which also affected the compositional analysis of the solid.

**Figure 5:**
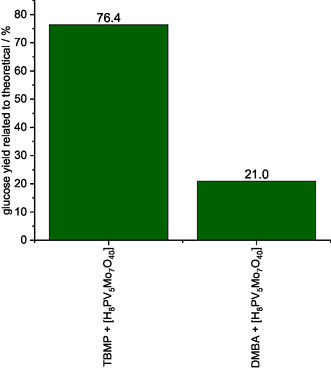
Enzymatic saccharification yield of processed cellulose‐rich solid using different IL/MeOH solvents and HPA‐5 as a catalyst analyzed via LAP NREL/TP‐5100‐63351.^[^ ^51^ ^]^ Reaction conditions: 120 °C, 24 h, 1000 rpm, 30 bar oxygen at reaction temperature, 10 g solvent (either methanolic TEA, methanolic DMBA, or methanolic TBMP), 0.5 g beech wood, 0.180 g HPA‐5 (1 mmol g^−1^ catalyst to solvent).

The cellulose‐enriched solid of the methanolic TBMP + HPA‐5 solvent resulted in a higher glucose yield after enzymatic saccharification compared to the cellulose‐enriched solid of the methanolic DMBA + HPA‐5 solvent (**Figure** **5**). However, addition of the redox‐active HPA‐5 catalyst slightly reduced the glucose yield after fractionation with the methanolic TBMP + HPA‐5 solvent to 76.4%, compared to 87.6% after fractionation using the solvent without catalyst. For the methanolic DMBA + HPA‐5 solvent, addition of the redox‐active HPA‐5 catalyst had an even more drastic effect, resulting in only 21% glucose yield after saccharification, significantly lower than the 76.8% yield obtained without the HPA‐5 catalyst. This effect might be related to interference from the precipitated catalyst.

### 2.3 Selection of a Redox‐Balanced POM Catalyst for the Enhanced POM‐Ionosolv Concept

To introduce lower redox activity while maintaining the Brønsted acidity of HPA‐5, other redox‐active metals, such as manganese (Mn), can be introduced into the POM structure, or a lower vanadium content than the reference system can be used. The catalysts surveyed in this study in this regard were various Keggin‐type structured POMs: HPA‐2, a twofold vanadium‐substituted POM (H_5_PV_2_Mo_10_O_40_); HPA‐VMn, a vanadium‐ and manganese‐substituted POM (H_8_PVMnMo_10_O_40_); HPA‐Mn2, a twofold manganese‐substituted POM (H_11_PMn_2_Mo_10_O_40_); and HPA‐Mn, a onefold manganese‐substituted POM (H_7_PMnMo_11_O_40_). The order of the catalysts in terms of redox activity is HPA‐5 > HPA‐2 > HPA‐VMn > HPA‐Mn2 > HPA‐Mn,^[^ ^36^ ^]^ as highlighted by square wave voltammetry (SWV) for the different catalysts (Figure S5, Supporting Information). Every peak in SWV represents a redox pair of the catalyst, and a larger number of peaks indicates higher redox activity. The higher the voltage of the pair, the higher the redox potential. It is not yet understood which pair(s) are responsible for the biomass oxidation in these POMs and clarifying this would exceed the scope of this study.

**Figure 6:**
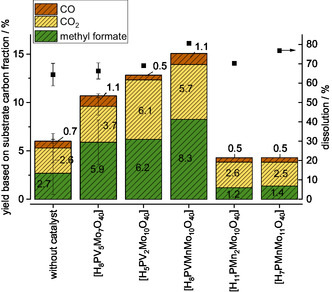
Product yields for the oxidation of beech wood in DMBA/MeOH solvent using HPA‐5 (H_8_PV_5_Mo_7_O_40_), HPA‐2 (H_5_PV_2_Mo_10_O_40_), HPA‐VMn (H_8_PVMnMo_10_O_40_), HPA‐Mn2 (H_11_PMn_2_Mo_10_O_40_), and HPA‐Mn (H_7_PMnMo_11_O_40_) as catalyst. Reaction conditions: 120 °C, 24 h, 1000 rpm, 30 bar oxygen at reaction temperature, 10 g methanolic DMBA solvent, 0.5 g beech wood, 0.180 g catalyst (1 mmol g^−1^ catalyst to solvent).

Like the HPA‐5 catalyst, the alternative V‐ and Mn‐containing POM catalysts were tested for solvent stability and formation of methyl formate under typical reaction conditions. All catalysts were also tested about their stability in the respective solvent system and found to be stable. This can be seen in the fourier transformed infrared spectroscopy (FT‐IR) spectra after the reaction showing the typical Keggin‐POM bands (see Figure S7, S9, and S11, Supporting Information). As shown in Figure S6, S8, and S10, Supporting Information, the methanolic TEA solvent was also unstable under reaction conditions with the alternative catalysts, despite their lower redox activity. Therefore, the methanolic TEA solvent was excluded from further studies. Instability was also observed in the methanolic DMBA and TBMP solvents under reaction conditions with highly redox‐active POMs, like HPA‐5 and HPA‐2 (Figure S8 and S10, Supporting Information). However, certain IL‐catalyst systems with less redox‐active POMs were identified as stable (HPA‐VMn, HPA‐Mn2, HPA‐Mn) as they do not show any methanol oxidation under reaction conditions, hence, these systems were studied further. For the methanolic DMBA solvent, the liquid‐ and gas‐phase analysis of the catalyst screening is shown in **Figure** **6**. The highest yield of methyl formate (8.3%) was obtained with the HPA‐VMn catalyst, which has medium redox activity. This catalyst contains vanadium as a highly redox‐active metal and manganese to reduce the redox activity.^[^ ^36^ ^]^ For the biomass fractionation, no clear trend was identified with the different alternative catalysts, but the most extensive dissolution (>70%) was achieved with the methanolic DMBA solvent containing the HPA‐VMn and HPA‐Mn catalysts.

**Figure 7:**
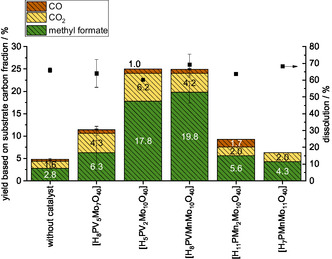
Product yields for the oxidation of beech wood in TBMP/MeOH solvent using HPA‐5 (H_8_PV_5_Mo_7_O_40_), HPA‐2 (H_5_PV_2_Mo_10_O_40_), HPA‐VMn (H_8_PVMnMo_10_O_40_), HPA‐Mn2 (H_11_PMn_2_Mo_10_O_40_), and HPA‐Mn (H_7_PMnMo_11_O_40_) as catalyst. Reaction conditions: 120 °C, 24 h, 1000 rpm, 30 bar oxygen at reaction temperature, 10 g methanolic TBMP solvent, 0.5 g beech wood, 0.180 g catalyst (1 mmol g^−1^ catalyst to solvent).

**Figure** **7** presents the product yields obtained from the catalyst screening with the methanolic TBMP solvent. No clear trend in dissolution (ranging from 60% to 69%) was observed with respect to the redox activity of the catalysts. However, the highest methyl formate yield of 19.8% was achieved with the HPA‐VMn catalyst in the methanolic TBMP solvent, which is more than double the yield obtained in the methanolic DMBA solvent. The HPA‐2 catalyst also produced a good methyl formate yield (17.8%) in the methanolic TBMP solvent, but the solvent was unstable under reaction conditions (Figure S10, Supporting Information), and the product‐to‐waste ratio (methyl formate vs. CO_2_) was lower (71.2%) compared to the HPA‐VMn catalyst (79.5%). Therefore, the HPA‐VMn catalyst and the methanolic TBMP solvent were identified as the best combined system for the alcoholic POM‐Ionosolv concept, and all further investigations proceeded with this combination. However, an inorganic precipitate was identified on every cellulose‐rich solid, regardless of the catalyst used which will be investigated in detail in the next section.

For a better comparison the turnover frequency (TOF) for methylformate formation using various POM catalysts in the different ILs DMBA and TBMP is shown in **Table** **2**. The TOF confirms that the most efficient tested reaction media is TBMP with the HPA‐VMn as catalyst (TOF = 3.23 × 10^−4^ s^−1^).

**Table 2:** Comparison of TOF for methyl formate as single valuable catalytic product using various catalysts in the different reaction media of DMBA and TBMP.

Catalyst	DMBA solvent TOF [s^−1^]	TBMP solvent TOF [s^−1^]
HPA‐5	1.04 × 10^−4^	8.57 × 10^−5^
HPA‐2	1.12 × 10^−4^	2.67 × 10^−4^
HPA‐VMn	1.46 × 10^−4^	3.23 × 10^−4^
HPA‐Mn2	2.06 × 10^−5^	8.66 × 10^−6^
HPA‐Mn	2.47 × 10^−5^	4.07 × 10^−5^

### 2.4 Investigating Catalytic Behavior under Reaction Conditions

An inorganic precipitate was identified in every cellulose‐rich solid after oxidative fractionation, regardless of the catalyst used. Pictures of the catalysts before the reaction and of the precipitates after the reaction are shown in Figure S12, Supporting Information. The occurring color changes for the catalysts HPA‐5, HPA‐Mn, and HPA‐Mn2 can be explained by the color change of the redox‐active metal in the POM. The identified preferred catalyst HPA‐VMn and its precipitate were investigated further. The nature of the precipitate formed in the preferred methanolic HPA‐VMn/TBMP solvent was examined using multiple analytical techniques. First, the elemental composition of the HPA‐VMn catalyst before the reaction and the inorganic precipitate after the reaction (separated from the mixture without substrate) was compared using inductively coupled plasma optical emission spectroscopy (ICP‐OES) measurements (Table S1, Supporting Information). In the inorganic precipitate, the vanadium stoichiometry decreased by about 50%, and the phosphorus content increased fourfold when normalized to the Mo content, whereas manganese was not detected. The high phosphorus content indicates that the inorganic precipitate could be a combination of POM fragments and phosphonium cations from the IL.

**Figure 8:**
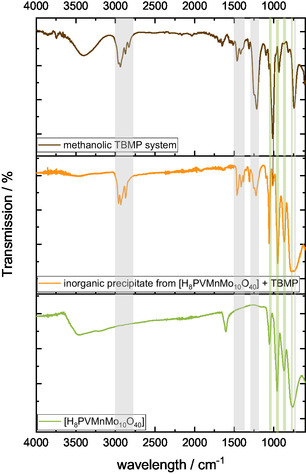
FT‐IR spectra of the methanolic TBMP solvent (top), the HPA‐VMn catalyst (H_8_PVMnMo_10_O_40_) (bottom), and the inorganic precipitate from HPA‐VMn in the TBMP/MeOH solvent (middle). The colored areas mark bands which appear similar in different spectra.

FT‐IR spectra of the inorganic precipitate were compared with the spectra of the initial catalyst HPA‐VMn and the methanolic TBMP solvent (**Figure** **8**). Characteristic bands for the Keggin‐type POM structure and the TBMP‐IL can be recognized (marked with colored areas). The Keggin‐type structure bands are located at 1055 cm^−1^ (P=O vibration), 954 cm^−1^ (M=O_t_ vibration), and 874 and 767 cm^−1^ (M—O—M vertex and edge vibration).^[^ ^36^ ^]^ The methanolic TBMP solvent bands are at 2938, 2961, 2878, 2835, 1458, 1418, 1250, 1209, 1008, 940, and 737 cm^−1^. This fingerprint was also identified by Albert et al.^[^ ^33^ ^]^ The bands at 2835 and 940 cm^−1^ could not be assigned, but the other bands of the methanolic TBMP solvent are evident in the FT‐IR spectrum of the inorganic precipitate, indicating that the precipitate is a salt with a Keggin‐type‐structured POM anion and likely the TBMP‐IL cation.

**Figure 9:**
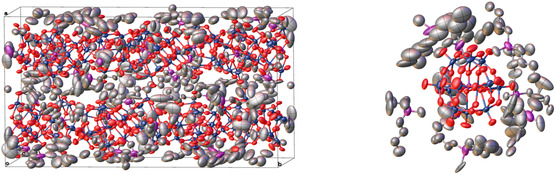
Solid‐state structure of the inorganic precipitate: unit cell (left) and single Keggin‐type anion surrounded by phosphonium cations (right). Color assignment: Metals (Mo, V, Mn) dark blue, phosphorus purple, oxygen red, carbon gray. Thermal ellipsoids are drawn at a probability level of 50%. Hydrogen atoms have not been modeled. The data for the structure is available through the joint Cambridge Crystallographic Data Centre and Fachinformationszentrum Karlsruhe Access Structures service (deposition number: 2422752)

A single‐crystal X‐ray diffraction (SCXRD) measurement of the precipitate reveals the molecular structure (**Figure** **9**), corroborating the composition suggested by FT‐IR spectroscopy and elemental analysis. The crystal structure consists of typical Keggin‐type POM structures composed of Mo and O atoms with a P atom as the central atom, surrounded by TBMP‐IL cations. The asymmetric unit contains three Keggin anions and nine phosphonium cations, with 12 formula units in the unit cell. The residual anionic charge is most likely compensated by protons, which were not modeled. Due to the highly disordered cation, detailed modeling of the elemental composition of the metal sites was not deemed useful as it does not significantly improve the model. The elemental distribution can be seen in the ICP‐OES data (Table S1, Supporting Information). Detailed information about the solid‐state structure is provided in Table S2 and S3, Supporting Information.

**Figure 10:**
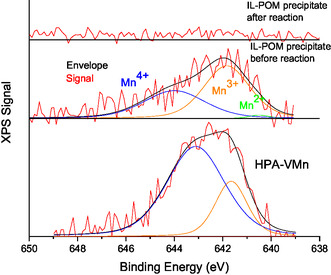
Comparison of the XPS signal of the Mn2p bands of the inorganic precipitate (IL‐POM precipitate) before and after the reaction and the initial HPA‐VMn POM catalyst. Reaction conditions: 120 °C, 24 h, 1000 rpm, 30 bar oxygen at reaction temperature, 10 g methanolic TBMP solvent, 0.180 g catalyst HPA‐VMn (1 mmol g^−1^ catalyst to solvent).

**Figure 11:**
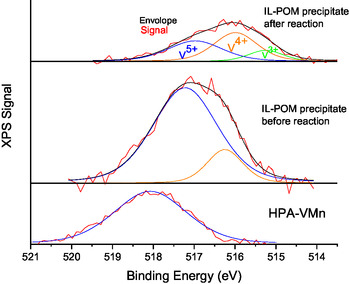
Comparison of the XPS signals of the V 2p bands for the inorganic precipitate (IL‐POM precipitate) before and after the reaction and for the initial HPA‐VMn POM catalyst. Reaction conditions: 120 °C, 24 h, 1000 rpm, 30 bar oxygen at reaction temperature, 10 g methanolic TBMP solvent, 0.180 g catalyst HPA‐VMn (1 mmol g^−1^ catalyst to solvent).

The valence charge of Mn and V ions plays a major role in the bond‐breaking and oxidation processes. Therefore, X‐ray photoelectron spectroscopy (XPS) was used to determine the electronic structure of the surface elements (Mn, V, and Mo) of the HPA‐VMn catalyst and the inorganic precipitate before and after a blank reaction (**Figure** **10** and **11**, Figure S13, and Table S4, Supporting Information). Three peaks were observed and fitted for the Mn 2p_3/2_ band for the HPA‐VMn catalyst and the inorganic precipitate prior to reaction, with binding energies around 641.72, 643.56, and 645.7 eV corresponding to Mn^2+^, Mn^3+^, and Mn^4+^ species, respectively.^[^ ^37^, ^38^, ^39^ ^]^ After the blank reaction (heating the reaction mixture up to 120 °C), manganese was not detected in the inorganic precipitate, which aligns with the ICP‐OES analysis results which showed no detectable amount of Mn after the reaction. Therefore, the manganese might dissolve into the liquid phase of the methanolic TBMP solvent during the reaction. Mixed Mn valence states were likely present during the redox reaction, as the interconversion between Mn^2+^, Mn^3+^, and Mn^4+^ enables efficient electron transfer between the reactants and the catalyst, thereby enhancing catalytic activity (see Figure 10).^[^ ^37^, ^38^, ^39^ ^]^ Interestingly, the manganese seems to have been present in different redox‐active states even before any possible reaction of the catalyst.

In contrast, the vanadium ions on the surface of the unused catalyst showed a single peak at a binding energy of 517 eV, fitted from the V 2p_3/2_ bands and assigned to V^5+^ species.^[^ ^40^ ^]^ During the formation of the inorganic precipitate, the vanadium was reduced to V^4+^ (peak at binding energy of 516 eV fitted from the V 2p_3/2_ band). After the blank reaction, the inorganic precipitate shows a third peak at around 515 eV, fitted from the V 2p_3/2_ band, which can be correlated to a V^3+^ species. This indicates that during the blank reaction, a redox reaction took place (see Figure 11), which might be the consecutive degradation of MeOH to methyl formate, as previously established. Although the end point of the reaction pathway of methyl formate and CO_2_ could not be detected via ^13^C‐NMR spectroscopy (Figure S10, Supporting Information), the surface of the inorganic precipitate shows a redox reaction of vanadium.

The methanolic TBMP solvent containing the HPA‐VMn catalyst was yellow, while the methanolic TBMP solvent alone was colorless. Figure S14 and S15, Supporting Information, shows ^31^P and ^51^V NMR spectra of the reaction solution and the methanolic TBMP solvent, as well as NMR spectra of the removed catalyst and the inorganic precipitate after dissolution in deuterated acetonitrile. The ^31^P NMR spectrum of the liquid reaction solution reveals the presence of a characteristic signal for the POM at −3.80 ppm and two small signals at −3.65 and −3.60 ppm, suggesting that not the entire POM catalyst was precipitated. The ^31^P NMR spectrum of the dissolved inorganic precipitate displays a TBMP‐IL‐specific signal at 31.8 ppm,^[^ ^41^ ^]^ as well the same signals of the POM, just slightly shifted downfield by 0.4 ppm, thereby substantiating a cation exchange between the IL and the POM. The ^51^V NMR spectra of the different phases show the presence of distinct vanadium species. In the methanolic TBMP solvent, the vanadium species exhibited peaks at −459 ppm (major) and at −539 ppm (minor), whereas the initial HPA‐VMn dissolved in deuterated acetonitrile exhibits one prominent peak at −554 ppm and smaller peaks between −568 and −556 ppm. The inorganic precipitate displayed only one prominent peak at −525 ppm and a small peak at −539 ppm. The latter peak was also present in the spectrum of the liquid reaction solution. Keggin‐type POMs are known to isomerize, and these isomers can be attributed to different signals. For a mono‐substituted vanadium POM, all positions in the Keggin framework are equivalent, and therefore only one signal occurs.^[^ ^36^, ^42^ ^]^ The shift of the prominent peak shows that the vanadium atom was situated in a different environment than that observed in the initial HPA‐VMn catalyst, suggesting that different catalyst species were present.

To determine whether the catalytic reaction was homogeneous or heterogeneous, the methanolic TBMP solvent with HPA‐VMn was heated in a sealed glass pressure tube and observed during the heating process. A comparison of the reaction solvent at room temperature with that at 95 °C is provided in Figure S16, Supporting Information, and differential scanning calorimetry (DSC) measurements showing a transition around 85 °C for the POM‐IL precipitate in shown in Figure S17, Supporting Information. At the elevated temperature, the inorganic precipitate completely dissolved in the reaction solution, resulting in a homogeneous system. Therefore, the reaction system of the methanolic POM‐Ionosolv concept with the TBMP‐IL and HPA‐VMn can be classified as a homogeneous reaction.

**Figure 12:**
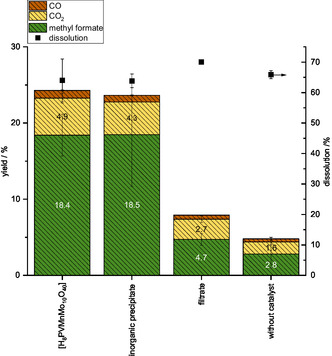
Product yields obtained from fractionative beech wood oxidation using different states of the HPA‐VMn catalyst: The filtered methanolic TBMP solvent where the HPA‐VMn catalyst is dissolved, the mixture of the methanolic TBMP solvent and the HPA‐VMn, and a previously precipitated inorganic precipitate in the methanolic TBMP solvent. For comparison reasons, the reaction without any catalyst is shown as well. Reaction conditions: 120 °C, 24 h, 1000 rpm, 30 bar oxygen at reaction temperature, 10 g methanolic TBMP solvent, 0.180 g HPA‐VMn or inorganic precipitate (1 mmol g^−1^ catalyst to solvent).

**Figure** **12** illustrates the catalytic performance of the various states of the HPA‐VMn catalyst. A reaction solution in which the HPA‐VMn was weighed into the methanolic TBMP solvent, as with other reactions presented previously (HPA‐VMn), is compared to a reaction solution in which the inorganic precipitate was used as a catalyst (inorganic precipitate). Moreover, a solution of the methanolic TBMP solvent that had been previously filtered to remove any inorganic precipitate was used as a reaction solution (filtrate). Additionally, a reaction conducted without a catalyst is illustrated in the figure (without catalyst). Figure S18, Supporting Information, explains the different experiments and shows the catalysts in pictures for a better understanding. As the yield of methyl formate nearly doubled in the filtrate compared to the reaction without a catalyst, it can be inferred that the amount of catalyst dissolved in the methanolic TBMP solvent at room temperature had significant catalytic activity. The reaction involving the HPA‐VMn catalyst and the inorganic precipitate as the catalyst exhibited a strikingly similar performance profile. Accordingly, it can be concluded that the inorganic precipitate can be reused as a catalyst. Further studies on the recycling of the catalyst may be required to ascertain the performance after several reactions.

### 2.5 Time Dependent Optimization Study for the Enhanced POM‐Ionosolv Concept

**Figure 13:**
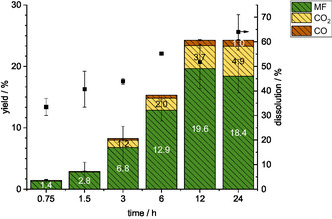
Product yields for fractionative beech wood oxidation with different reaction times. Reaction conditions: 120 °C, 24 h, 1000 rpm, 30 bar oxygen at reaction temperature, 10 g methanolic TBMP solvent, 0.180 g HPA‐VMn (1 mmol g^−1^ catalyst to solvent).

To optimize the formation of the methyl formate product, a time course study was performed for the reaction with the HPA‐VMn catalyst in the methanolic TBMP system. The analysis of the gas and liquid phases is illustrated in **Figure** **13**. Regarding the production of methyl formate, the results indicate that a reaction time of 12 h was favorable, as the product yield declined with a longer reaction of 24 h, during which the CO_2_ yield concurrently increased to 4.9%. The yield of CO remained ≤1% at all reaction times, and the dissolution gradually increased to 64% over the 24 h period.

To evaluate the efficacy of a reaction time of 12–24 h, the cellulose‐enriched solid was also characterized (Figure S19 and S20, Supporting Information). Compositional analysis showed that 92.1% and 91.3% glucan was obtained in the solids after 12 and 24 h, respectively. However, the enzymatic saccharification increased by around 40% between 12 and 24 h, making the reaction time of 24 h preferable. Extending the reaction time would lower the selectivity and the methyl formate yield even further and was therefore not tested.

### 2.6 Separation and Purification of Reaction Products for the Enhanced POM‐Ionosolv Concept

#### 2.6.1 Processed Solid

Due to the presence of precipitated catalyst on the cellulose‐enriched solid, a washing procedure was established to remove the precipitate. The established washing procedure^[^ ^17^, ^18^ ^]^ used the volatile cosolvent (MeOH) to wash off nonvolatile IL and catalyst from the cellulose‐enriched solid. However, this led to a high amount of vanadium on the solid as shown in Table S5, Supporting Information. Molybdenum is the main metallic component in the catalyst, and therefore, a good indicator to check for the presence of catalyst precipitate on the cellulose‐enriched solid. The other elements of the catalysts were also measured with ICP‐OES, and the results show a similar result for the molybdenum content (Table S5, Supporting Information). The molybdenum content in the cellulose‐enriched solid after washing with MeOH was determined to 14.4% by ICP‐OES. Hence, a second washing step was investigated utilizing dimethylsulfoxide (DMSO) or acetone, as both solvents are expected to dissolve the catalyst precipitate without interfering with the cellulose‐enrich solid. Importantly, DMSO could reduce the amount of molybdenum on the cellulose‐enriched solid below the detection limit (0.03%), whereas acetone washing reduced the amount of molybdenum to 0.06%.

**Figure 14:**
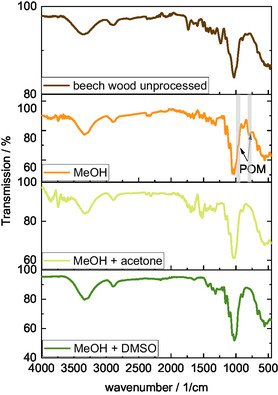
Comparison of different FT‐IR spectra recorded of unprocessed beech wood and cellulose‐enriched solid washed with methanol (MeOH), methanol and acetone (MeOH + acetone), and methanol and dimethyl sulfoxide (MeOH + DMSO).

The Keggin‐type structure of the HPA‐VMn catalyst was also visible in the FT‐IR spectrum after the first MeOH wash (marked in **Figure** **14** with POM) with characteristic bands at 1059 cm^−1^ (P—O vibration), 962 cm^−1^ (M=O_t_ vibration), and 877 and 744 cm^−1^ (M—O—M vibrations), even though the former two bands overlapped with bands from the cellulose‐enriched solid. As expected, the characteristic bands were not detected after the second washing step, confirming the effectiveness of the overall pulp washing procedure. The recycling of the catalyst from the washing solutions was not investigated in this study, potential methods are distillation or precipitation from the washing solution.

**Figure 15:**
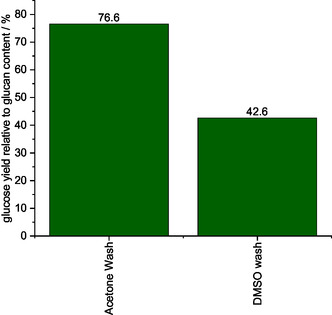
Comparison of enzymatic saccharification of the processed cellulose‐enriched solid washed with acetone and DMSO analyzed via LAP NREL/TP‐5100‐63351.^[^ ^51^ ^]^ Reaction conditions: 120 °C, 24 h, 1000 rpm, 30 bar oxygen at reaction temperature, 10 g methanolic TBMP solvent, 0.5 g beech wood, 0.180 g HPA‐VMn (1 mmol g^−1^ catalyst to solvent).

One disadvantage of DMSO is its high boiling point, which makes distillation of the solvent energy intensive. Therefore, the cellulose‐enriched solid was contaminated with DMSO after washing, necessitating a third washing step to remove DMSO. To emphasize this, comparative CHNS analysis was performed on the washed solids, and the measured sulfur contents were compared. The sulfur content after the first MeOH washing was 0.27 and 0.23 wt% after the second washing with acetone. In contrast, the sulfur content increased to 30.43 wt% after washing with DMSO (Table S5, Supporting Information). The corresponding compositional analysis data of the two cellulose‐enriched solids are shown in Figure S21, Supporting Information. The glucan content of both samples was above 90% (91.3% after acetone washing, 94.5% after DMSO washing), so no preference can be derived from the compositional analysis. Therefore, the two cellulose‐enriched solids were evaluated using enzymatic saccharification. The residual DMSO in the cellulose‐enriched solid significantly influenced the yield of glucose from enzymatic saccharification, resulting in a yield (42.6%) that was around 30% lower than that of the acetone‐washed solid (76.6%) (**Figure** **15**). This lower performance might be explained by the suppression of endo‐1,4‐β‐glucosidase in the cellulase mixtures.^[^ ^43^ ^]^ Thus, the preferred washing agent for the second washing procedure was acetone, and this was used in further analysis.

#### 2.6.2 Liquid Reaction Products

After purifying the cellulose‐enriched solid, the separation of the liquid phase product methyl formate was investigated. An ex situ distillation in a round‐bottom flask was performed to test the separation of methyl formate from the solvent, preferably from the IL. Methyl formate has a boiling point of 32 °C and MeOH of 65 °C.^[^ ^44^, ^45^ ^]^ As the methyl formate had a moderate concentration in the experiments, it proved beneficial to codistill the methyl formate and the MeOH at the boiling temperature of MeOH. The distillation allowed for the recovery of 96% of methyl formate with only MeOH as the solvent in the head, while the IL remained in the flask. ^13^C NMR spectra of the two distillation fractions obtained from a reaction solution (Figure S22 and S23, Supporting Information) confirm that distillation is a useful method for isolating the desired methyl formate product.

### 2.7 Substrate Scope for the Enhanced POM‐Ionosolv Concept

**Figure 16:**
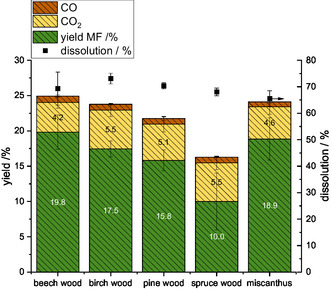
Product yields and dissolution of different types of lignocellulosic biomass as substrate using the methanolic TBMP solvent. Reaction conditions: 120 °C, 24 h, 1000 rpm, 30 bar oxygen at reaction temperature, 10 g methanolic TBMP solvent, 0.180 g HPA‐VMn (1 mmol g^−1^ catalyst to solvent).

The ionosolv‐fractionation can be affected by the type of lignocellulosic biomass.^[^ ^17^, ^20^, ^46^ ^]^ To evaluate this effect, the substrate scope was investigated using birch wood as a second hardwood, pine and spruce wood as representative softwoods, and miscanthus as grass biomass. The focus was on the yield of methyl formate, the glucan content in the cellulose pulp, and glucose yield obtained with enzymatic saccharification. **Figure** **16** presents the yields of the liquid phase product methyl formate and the gaseous phase products CO_2_ and CO, as well as the dissolution of the substrate. A variation in the yield of methyl formate between 10.0%–19.8% was found, with the hardwoods and miscanthus showing higher yields (17.5% birch, 18.9% miscanthus, 19.8% beech) than the softwoods (15.8% pine, 10.0% spruce). Conversely, the yield of CO_2_ (4.2%–5.5%) and CO (≈1%) did not differ much between substrates. The substrate dissolution in the methanolic TBMP‐IL solvent was also similar (between 60% and 70%), with no recognizable trend between the substrate types.

**Figure 17:**
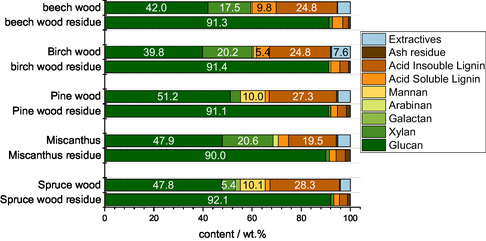
Compositional analysis of different types of lignocellulosic biomass and the processed cellulose‐enriched solid done via NREL/TP‐510‐42618.^[^ ^47^ ^]^ Reaction conditions for preparing the solids: 120 °C, 24 h, 1000 rpm, 30 bar oxygen at reaction temperature, 0.5 g beech wood, 10 g methanolic TBMP solvent, 0.180 g HPA‐VMn (1 mmol g^−1^ catalyst to solvent). ASL = acid soluble lignin, AIL = acid insoluble lignin.

**Figure 18:**
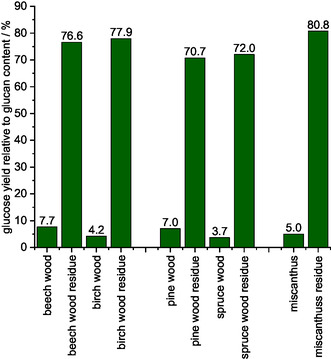
Enzymatic saccharification yield for different lignocellulosic biomass types compared to enzymatic saccharification of cellulose‐rich solids generated by using methanolic POM ionosolv. Reaction conditions: 120 °C, 24 h, 1000 rpm, 30 bar oxygen at reaction temperature, 10 g methanolic TBMP solvent, 0.5 g beech wood, 0.180 g HPA‐VMn (1 mmol g^−1^ catalyst to solvent).

**Figure** **17** shows the composition of the different feedstock pulps compared to the composition of the original biomass. All cellulose‐enriched solids obtained after the POM‐Ionsolv concept with the methanolic TBMP solvent had a glucan content of >90%, and the cellulose yield for all substrates was >60%, with the highest yield (73%) achieved with miscanthus. Detailed data can be found in Figure S24, Supporting Information. For beech wood as an example this would mean: From 1 g of beechwood, 0.67 g of cellulose and 0.07 g of methyl formate can be produced meaning a high utilization of the whole biomass, only 0.01 g are not valorized as gaseous products (CO and CO_2_). The glucose yield after enzymatic saccharification of the cellulose‐enriched solids from different substrates were >70%, as shown in **Figure** **18**. Even the treated miscanthus solid achieved a glucose yield of 80.8%, proving the versatility of the updated POM‐Ionosolv 2.0 concept using a methanolic TBMP‐IL solvent and HPA‐VMn for various types of biomass.

## 3 Conclusions

Production of high methyl formate yields of 20% from beech wood and a cellulose‐enriched solid with a glucan content exceeding 90% via an improved POM‐Ionosolv concept has been demonstrated by utilizing MeOH as a co‐solvent and identifying a redox‐balanced HPA‐VMn‐POM catalyst. Different MeOH/IL (70 wt%/30 wt%) solvent mixtures were employed for the fractionation of the lignocellulosic feedstock, while simultaneously oxidizing the dissolved compounds to methyl formate via the homogeneous POM catalyst. The study identified the phosphonium‐based IL [TBMP][MeSO_4_] as the most suitable IL for the conversion. The cellulose‐enriched solid retained 77% of the glucan contained in the beech biomass for subsequent enzymatic saccharification. Furthermore, it was demonstrated that the IL/MeOH reaction media is stable against oxidation under the utilized reaction conditions. A catalyst screening of POMs with various redox activity identified HPA‐VMn (H_8_PVMnMo_10_O_40_) as the optimal catalyst for achieving the highest yield of methyl formate (≈20%). A comprehensive examination of the interactions between the catalyst and IL revealed that an inorganic POM‐IL salt, which serves as a homogeneous catalyst in the system under operating conditions, precipitated out at room temperature. The POM‐IL salt structure was elucidated through comprehensive analysis employing SCXRD, XPS, and FT‐IR spectroscopy and ICP‐OES.

To the best of our knowledge, this is the first instance of such a POM‐IL structure being characterized via SCXRD measurements. With an optimal reaction time of 24 h, HPA‐VMn in [TBMP][MeSO_4_] enabled dissolution of 70% of beech wood in the liquid phase, representing an optimal compromise between the usability of cellulose‐enriched solid and the yield of methyl formate. The separation of methyl formate from the IL and catalyst via distillation was demonstrated, and an optimized washing procedure for the purification of the cellulose‐enriched solid with MeOH and acetone was established. Importantly, the catalytic system also proved effective for the conversion of softwood (pine and spruce) and grass biomass (miscanthus), with yields of 10% (spruce) to 19% (miscanthus) of methyl formate. All cellulose‐enriched solids exhibited a glucan content exceeding 90% and an enzymatic saccharification accessibility exceeding 70%. In these pulps, over 60% of the cellulose in the initial substrates could be retained in the cellulose pulp, with 73% of the cellulose content retained for Miscanthus.

In conclusion, the previous water‐based POM‐Ionosolv concept has been enhanced by the introduction of MeOH as a co‐solvent and the incorporation of a novel phosphonium‐based IL, along with a new redox‐balanced POM catalyst with a redox activity tuned for the application. Additionally, the catalyst has been subjected to comprehensive characterization through a range of analytical techniques. With further optimization of the catalyst recycling process, this system demonstrates high potential for application in biorefineries.

## 4 Experimental Section

4.1

4.1.1

##### Chemicals

All chemicals and feedstocks are commercially available and were used without further purification. The chemicals used are shown in **Table** **3** with their purity and supplier.

**Table 3:** Used chemicals with purity and supplier.

Chemical	Supplier	Purity
Beech wood	Mushrooms and equipment shop	–
Miscanthus	Silwood Park campus (Imperial College London, UK).	–
Birch wood	Räucherspan.de (URL: https://www.raeucherspan.de/)	–
Spruce wood	Räucherspan.de (URL: https://www.raeucherspan.de/)	–
Pine wood	Jbach GmbH	–
Methanol	VWR chemicals	>99.5%
Acetone	Walter cMP GmbH & Co. KG	Pure
Dimethylsulfoxide (DMSO)	Grüssing GmbH	99.5%
Tributylmethylphosphonium methyl sulfate ([TBMP][MeSO_4_], TBMP)	Iolitec Ionic Liquids Technologies GmbH	>95%
*N,N*‐Dimethylbutylammonium hydrogen sulfate ([DMBA][HSO_4_], DMBA)	Synthesized at Imperial College London, UK according to literature^[^ ^52^ ^]^	–
Triethylammonium hydrogen sulfate ([TEA][HSO_4_], TEA)	Synthesized at University of Hamburg according to literature^[^ ^53^ ^]^	–
Polyoxometalate catalysts (H_8_PV_5_Mo_7_O_40_ (HPA‐5), H_5_PV_2_Mo_10_O_40_ (HPA‐2), H_8_PVMnMo_10_O_40_ (HPA‐VMn), H_11_PMn_2_Mo_10_O_40_ (HPA‐Mn2), H_7_PMnMo_11_O_40_ (HPA‐Mn1))	Synthesized according to literature^[^ ^22^, ^36^, ^54^, ^55^, ^56^ ^]^	–

##### Experimental Setup and Typical Working Method

All catalytic experiments were performed in 20 mL Hastelloy C276 autoclaves equipped with a magnetic stirrer. The catalyst (0.1 mmol), fractionated substrate (*x* ≤ 630 μm, 0.5 g), and 10 g of different IL/MeOH mixtures with a ratio of 30 wt%/70 wt% were loaded into the reactor. The reactor was then closed and purged twice with 20 bar of oxygen before being repressurized with oxygen to get 20 bar pressure at reaction temperature. While the reactor was heated, the stirrer was set to 300 rpm, and at reaction temperature, the stirrer was adjusted to 1000 rpm. After the reaction time, the reactor was cooled to room temperature, and a gas sample was taken and analyzed via gas chromatography using a flame ionization detector (FID). The reactor content was filtered to separate the solid and the liquid phase. The solid was washed and analyzed via National Renewable Energy Laboratory (NREL) method, FT‐IR spectroscopy, and CHNS elemental analysis and subsequently processed via enzymatic saccharification. The liquid phase was analyzed for reaction products via ^1^H NMR spectroscopy with an internal standard of tert‐butanol and MeOD in a ratio of 1:5 with the sample. For additional analysis, ^13^C NMR, ^51^V NMR, and ^31^P NMR were performed.

##### Catalyst Characterization

The catalyst and the catalyst precipitate were analyzed via FT‐IR spectroscopy, ICP‐OES analysis, XPS, SCXRD, and XRD. The liquid phase containing the catalyst was analyzed via ^51^V NMR and ^31^P NMR. The catalyst was also analyzed via SWV.

##### Washing Procedure

The solid after the reaction was first washed with 50 mL MeOH as a standard procedure, followed by washing with 100 mL dimethyl sulfoxide (DMSO) or acetone.

##### Distillation

A classic distillation setup consisting of a four‐neck flask and a distillation bridge was initially used for distillation. A KPG stirrer from Heidolph (RZR 2021) was used for stirring. Water was used as a coolant for the distillation bridge. The four‐neck flask was heated with a heating block to 75 °C and stirred at 200 rpm. The temperature inside of the flask was measured to be 67 °C, corresponding to the boiling temperature of MeOH. The product flask (the head of the distillation) was cooled with an ice water bath. For the experiment, three different methanolic TBMP solvent experiments with HPA‐VMn as catalyst (reaction conditions: 120 °C, 24 h, 1000 rpm, 30 bar of oxygen at reaction temperature, 10 g solvent [TBMP][MeSO_4_]/MeOH 30 wt%/70 wt% (TBMP), 0.180 g HPA‐VMn (1 mmol g^−1^ catalyst), 20 mL Hastelloy autoclave) were combined into 30 mL of feed solution and distilled.

##### Analytics

The solid and the substrates were analyzed using a Euro Vector EA 3000 to determine the carbon (C), nitrogen (N), hydrogen (H), and sulfur (S) content. The FT‐IR spectroscopy was performed with an IR‐Spirit from Shimadzu, and all data were smoothed using an 80‐point Savitzky‐Golay smooth. The typical wavelengths for the Keggin‐type POM structure are: $v^{˜}$ [cm^−1^] = 3481 (w, O—H, H_2_O), 1603 (O—H, lattice H_2_O), 1055 (w, P—O), 959 (me, M=O_t_), 871 ((M—O—M)_vertex_), 762 ((M—O—M)_edge_).^[^ ^36^ ^]^ The compositional analysis was prepared according to the LAP NREL/TP‐510‐42618.^[^ ^32^ ^]^ The enzymatic saccharification was prepared according to the LAP NREL/TP‐5100‐63351.^[^ ^47^ ^]^

The gas phase was analyzed using a Varian GC 450 equipped with a Shin‐carbon column (2 m × 0.75 mm) and both a thermal conductivity as well as a FID.

SWV data were measured with a concentration of 1 mmol L^−1^ in acetonitrile and a scan rate of 5 mV s^−1^ on an Ivium Potentiostat. The working electrode was glassy carbon (diameter: 3 mm), the reference electrode was Ag/Ag^+^, and the counter electrode was platinum. All SWV measurements were done with a scan rate of 5 mV s^−1^, a modulation amplitude of 20 mV, and a frequency of 25 Hz. The elemental composition for Mn, Mo, P, and V was determined using a Solaar S Series ASS‐F (atom emission spectroscopy‐flame) from Thermo (Mn) and an ARCOS ICP‐OES from Spectro (V, P, Mo). The liquid phase after the reactions was analyzed via NMR spectroscopy. All NMR spectra were measured with a Bruker Avance III HD 600 MHz. The ^51^V NMR was recorded with the following parameters: number of scans 4096, receiver gain 2050, relaxation delay 0.5, and pulse width 11.13. The ^31^P NMR was recorded with the following parameters: number of scans 1024, receiver gain 1820, relaxation delay 1.0, and pulse width 11.2. The ^13^C NMR was recorded with the following parameters: number of scans 512, receiver gain 2050, relaxation delay 1.0, and pulse width 9.31. The ^1^H NMR was measured with a scan number of 16, a receiver gain of 2.8, a relaxation delay of 1.0, and a pulse width of 10.37.

XPS analyses were performed using a Thermo Scientific system at room temperature with AlKα radiation (1484.6 eV) and a spot size of 400 μm. A flood gun was used to reduce sample charging effects, and the obtained spectra were further corrected by setting the C1s binding energy at 284.8 eV. Data processing was done using the Avantage 4.87 software. Mn2p_3/2_ and V2p_3/2_ signals were each deconvoluted into three peaks using mixed Gauss–Lorentz functions (L/G: 30%–70% mix) using the Powell fitting algorithm. The peaks assigned to Mn^2+^, Mn^3+^, and Mn^4+^ were constrained to binding energies of 640.0 ± 0.5 eV, 641.5 ± 0.5 eV, and 643.5 ± 0.5 eV respectively, with their full‐width at half‐maximum (FWHM) allowed to vary between 1 and 3 eV. The peaks assigned to V^3+^, V^4+^, and V^5+^ were constrained to binding energies of 515.0 ± 0.25 eV, 516.0 ± 0.25 eV, and 517.0 ± 0.25 eV respectively, with their FWHM allowed to vary between 1 and 3 eV.

XRD measurements were carried out on a Huber G670 powder diffractometer using Cu Kα radiation within a 2θ range of 5°–70° in steps of 0.005° at a speed of 0.02°s^−1^.

For the SCXRD experiment, a suitable single crystal of C_39_Mo_12_O_40_P_4_ was selected and mounted on a SuperNova, dual source (Cu Kα X‐ray), Atlas diffractometer. The crystal was kept at 120.0(1) K during data collection. Using Olex2,^[^ ^48^ ^]^ the structure was solved with the SHELXT^[^ ^49^ ^]^ structure solution program using Intrinsic Phasing and refined with the SHELXL^[^ ^50^ ^]^ refinement package using Least Squares minimization. The crystal structure of C_39_Mo_12_O_40_P_4_ contains a highly disordered network of organic ligands. To get a complete overview of the ligand distribution in the structure, molecules were modeled with restraints and constraints: RIGU, SADI, DFIX, DANG, DELU, SIMU, and ISOR.

For DSC measurements, about 40 mg of sample was weighed into a Mettler Toledo DSC 1 and measured with a temperature program starting at −50 °C, followed by heating at 10 K min^−1^ to 150 °C with a final hold at 120 °C for 100 s.

##### Formulas for Calculation

The following equations show the formulas for the basic calculations done in this study. The molar amount of carbon (*n*_c_) in a sample was calculated via Equation (1) with *m* as mass, *w*_c_ as mass fraction of carbon measured via CHNS analysis, and *M*_c_ as molar mass of carbon.

(1) $$n_{C}=\frac{m\;*\;w_{C}}{M_{C}}$$

The amount of carbon fraction dissolved into the reaction media (*n*_CC,dissolved_) was determined via Equation (2).

(2) $$n_{\text{C,dissolved}}=n_{\text{C, substrate}}-n_{\text{C, solid residue}}$$

The dissolution of the carbon fraction (dissolution) is the amount of carbon not retained in the solid, which therefore was dissolved in the reaction solution. This is shown in Equation (3).

(3) $$\text{dissolution}=\left(1-\frac{m_{\text{solid residue after reaction}}\;*\;w_{\text{C, solid residue after reaction}}}{m_{\text{substrate, initial}}*w_{\text{C, substrate initial}}}\right)\;*\;100\%$$

As some solvents were susceptible to oxidation with some of the POM catalysts, a new molar amount of product i was introduced in Equation (4). This *n*_i,new_ is the difference between the amount of product i (i = MF, CO_2_, CO) found in the reaction with substrate (*n*_i,substrate_) and the amount of product i found in the reaction without substrate (blank reaction).

(4) $$n_{i,\text{new}}=n_{\text{i,substrate}}-n_{\text{i, blank}}$$

The yield of the products is calculated via Equation (5) using the new amount of product (*n*_i,new_) in relation to the amount of dissolved carbon fraction (*n*_C,dissolved_), as this is the only amount which can be converted into liquid and gaseous products.

(5) $$Y=\frac{n_{\text{i,substrate,new}}}{n_{\text{C,dissolved}}}*100\%$$

The value to waste ratio is calculated via Equation (6).

(6) $$\text{value to waste ratio}=\frac{Y_{\text{methyl formate}}}{Y_{\text{CO}}+Y_{{\text{CO}}_{2}}}$$

The TOF is calculated via Equation (7)

(7) $$\text{TOF}=\frac{n_{\text{methyl formate}}}{n_{\text{catalyst}}\;*\;t}$$

where *t* is the time of the reaction in seconds, *n*_catalyst_ is the molar amount of catalyst used, and *n*_methyl formate_ is the molar amount of methyl formate.

## Supporting Information

The supporting information is accompanied by various additional results and analytical data of catalysts and reaction solutions.

## Conflict of Interest

The authors declare no conflict of interest.

## Supporting information

Supplementary Material

## Data Availability

The data that support the findings of this study are available from the corresponding author upon reasonable request.
