# Supplementary material for: Boosting POM‐Ionosolv Biorefining of Lignocellulosic Biomass by Using Redox‐Balanced Polyoxometalate Catalysts in Methanolic Ionic Liquid Reaction Media
Source: ChemSusChem. 2025 Sep 10;18(20):e202501034. doi: 10.1002/cssc.202501034 (PMC12548942; doi:10.1002/cssc.202501034)
Supplement: Supplementary file 1 — Supplementary Material [file CSSC-18-e202501034-s001.pdf]

# Supporting Information

## Boosting POM-Ionosolv biorefining of lignocellulosic biomass by using redox-balanced polyoxometalate catalysts in methanolic ionic liquid reaction media

Stefanie Wesinger[a], Aleksandra Rabiner,[c] Suhaib Nisar[b,c], Leonhard Schill[d], Mariusz Grzegorz Kubus[d], Maximilian J. Poller[a], Anders Riisager[d], Agnieszka Brandt-Talbot[b], Jason P. Hallett[c] and Jakob Albert[a]*

[a] Institute of Technical and Macromolecular Chemistry, University of Hamburg, Bundesstraße 45, 20146 Hamburg, Germany

[b] Department of Chemistry, Imperial College London, Molecular Sciences Research Hub, White City Campus, United Kingdom

[c] Department of Chemical Engineering, Imperial College London, Bone Building, South Kensington Campus, United Kingdom

[d] Department of Chemistry, Technical University of Denmark, Kemitorvet, 207, 2800 Kgs. Lyngby, Denmark

**Contact details of the corresponding author: jakob.albert@uni-hamburg.de**

# Investigation to improve the material systems

## Morphology of processed solids

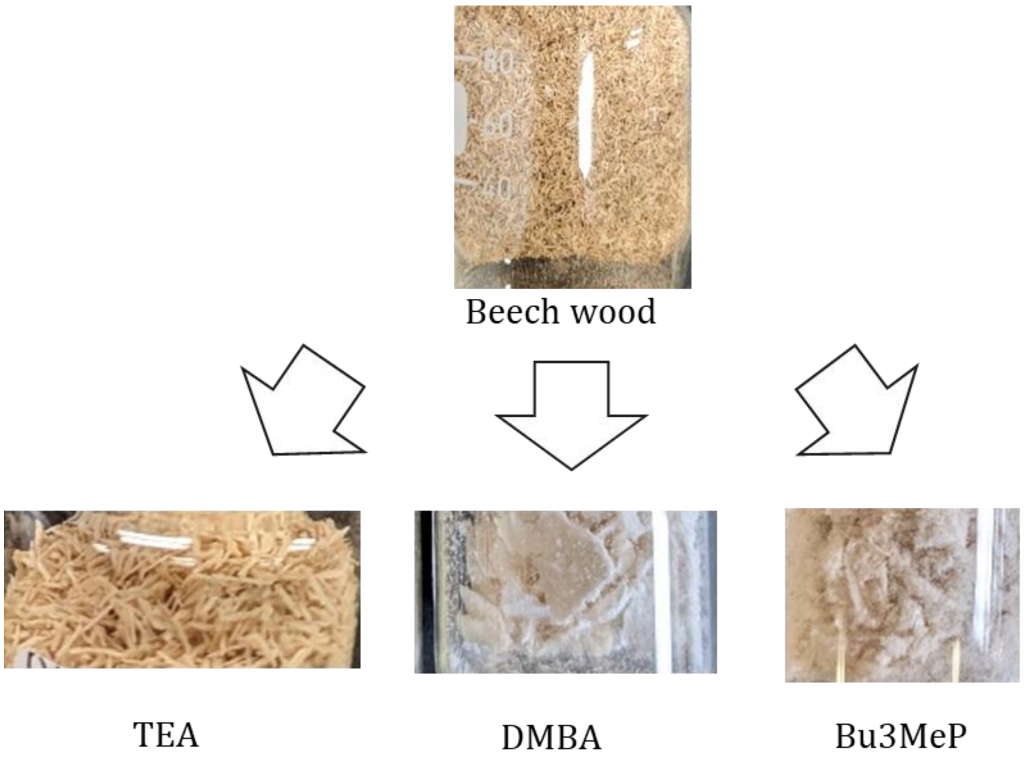


Figure S1: Pictures of beech wood and the processed solid residues with different IL/MeOH solvent systems to show the different morphologies. *Reaction conditions: 120 °C, 24 h, 1000 rpm, 30 bar of oxygen at reaction temperature, 10 g solvent ([TEA][$HSO_4$]/MeOH 30 wt.%/70 wt.% (TEA), [DMBA][$HSO_4$]/MeOH 30 wt.%/70 wt.% (DMBA), [TBMP][$MeSO_4$]/MeOH 30 wt.%/70 wt.% (TBMP)), 0.5 g beech wood.*

**Stability of methanolic ionic liquid solvent**

***Stability of TEA ([TEA][$HSO_4$]/MeOH 30 wt.%/70 wt.%.) with HPA-5***

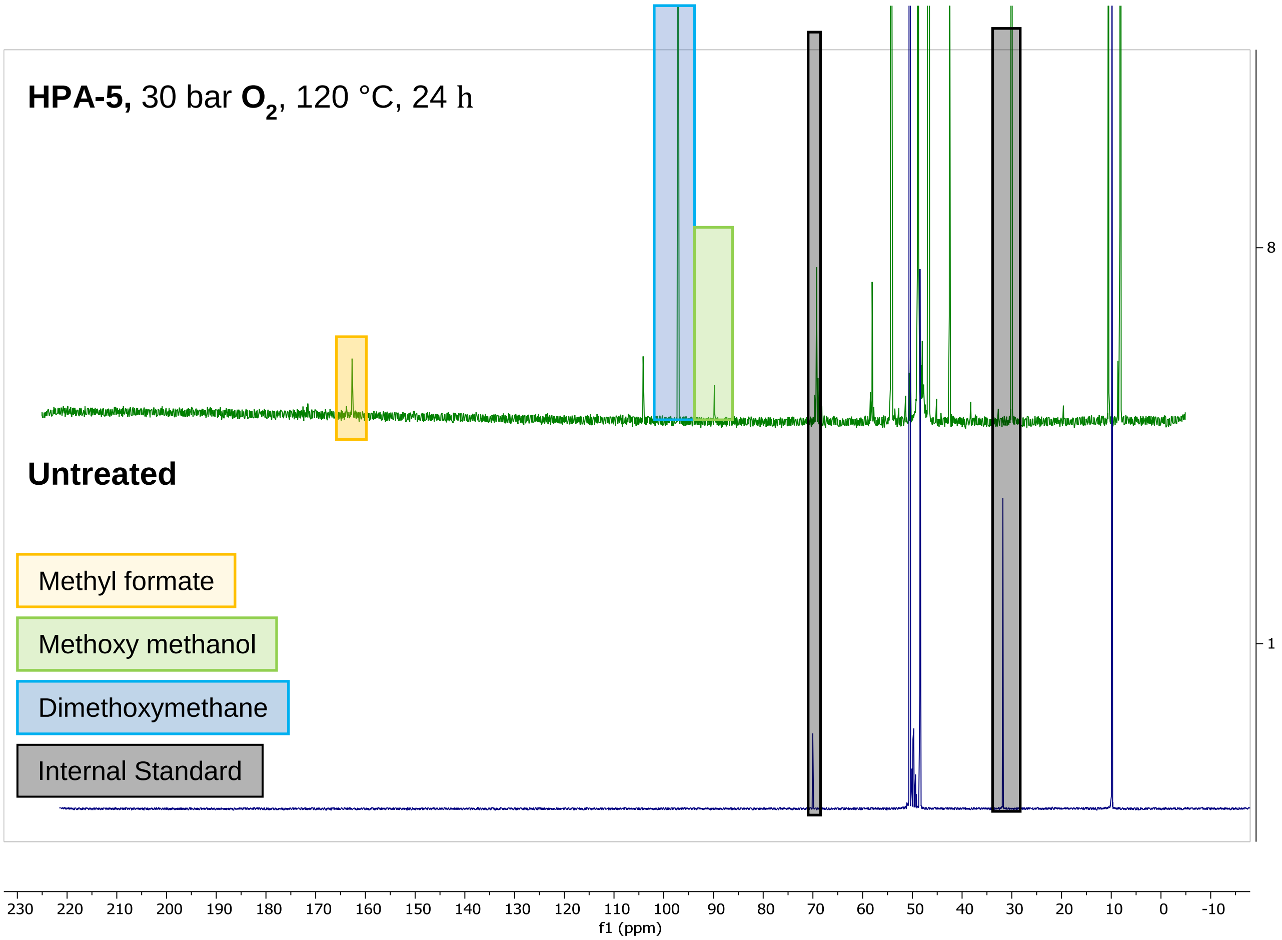


Figure S2: $^{13}C$ NMR spectra of untreated TEA and with HPA-5 under reaction conditions treated TEA. TEA is an abbreviation for the solvent mixture of [TEA][$HSO_4$]/MeOH 30 wt.%/70 wt.%. The identified methanol oxidation products (methyl formate (163 ppm), methoxy methanol (91 ppm, 56 ppm) and dimethoxymethane (98 ppm, 51 ppm) and the internal standard (tert-butanol) are marked.

***Stability of DMBA ($[DMBA][HSO_4]$/MeOH 30 wt.%/70 wt.%.) with HPA-5***

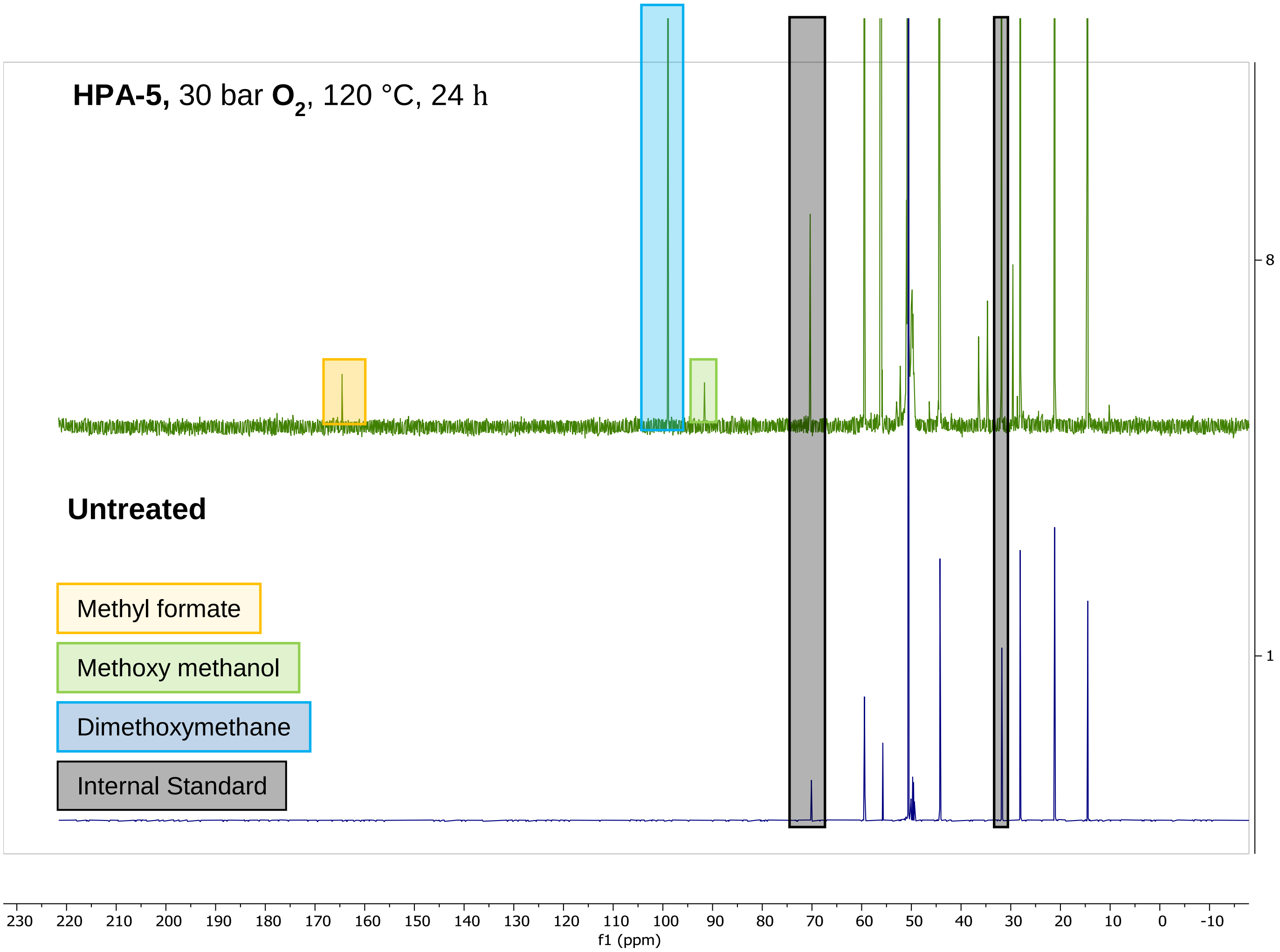


Figure S3: $^{13}C$ NMR spectra of untreated DMBA and with HPA-5 under reaction conditions treated DMBA. DMBA is an abbreviation for the solvent mixture of $[DMBAA][HSO_4]$/MeOH 30 wt.%/70 wt.%. The identified methanol oxidation products (methyl formate (163 ppm), methoxy methanol (91 ppm, 56 ppm) and dimethoxymethane (98 ppm, 51 ppm) and the internal standard (tert-butanol) are marked.

***Stability of TBMP ([TBMP][MeSO$_4$]/MeOH 30 wt.%/70 wt.%.) with HPA-5***

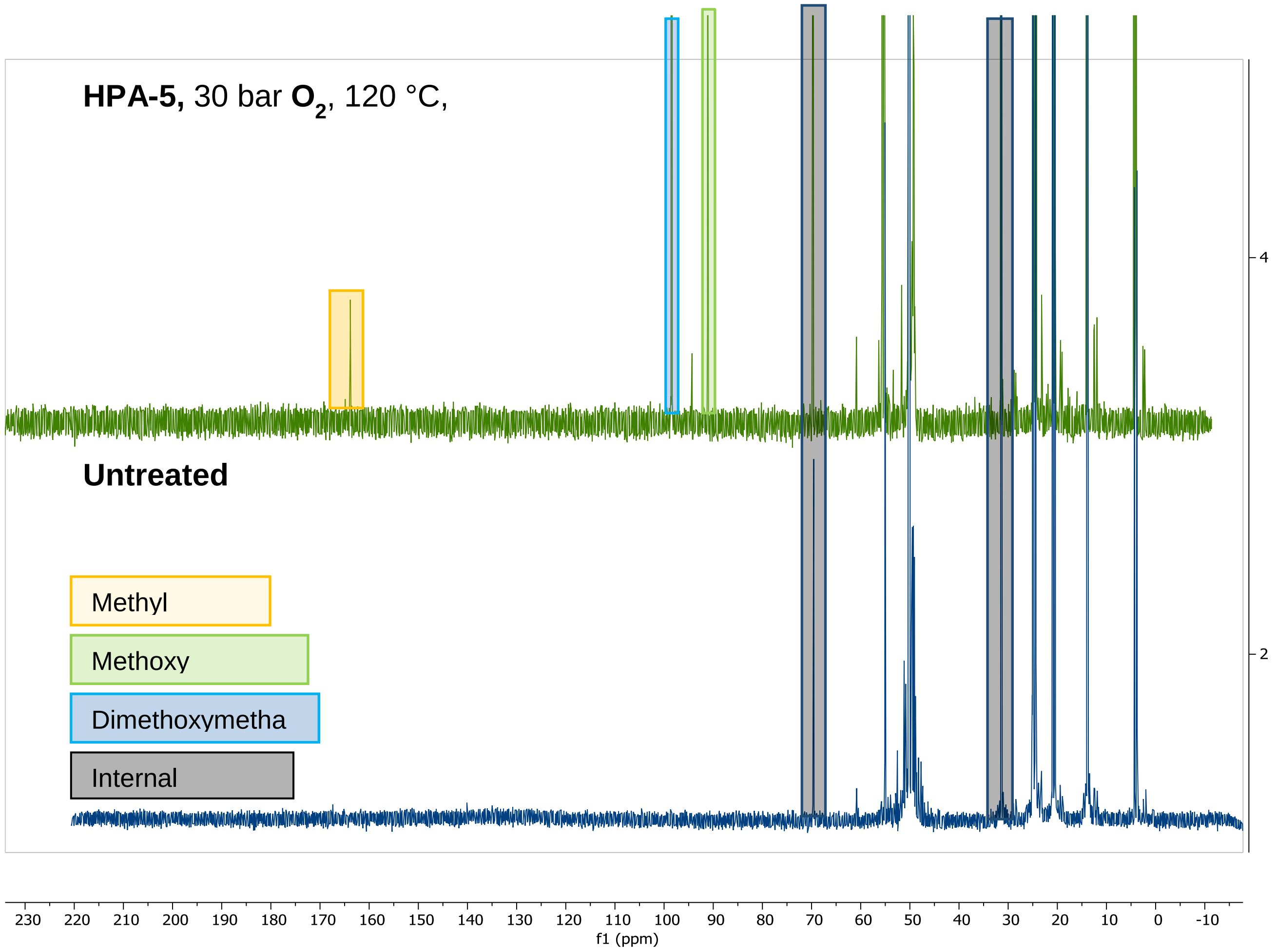


Figure S4: $^{13}$C NMR spectra of untreated Bu3MeP and with HPA-5 under reaction conditions treated Bu3MeP. Bu3MeP is an abbreviation for the solvent mixture of [Bu3MeP][MeSO$_4$]/MeOH 30 wt.%/70 wt.%. The identified methanol oxidation products (methyl formate (163 ppm), methoxy methanol (91 ppm, 56 ppm) and dimethoxymethane (98 ppm, 51 ppm) and the internal standard (tert-butanol) are marked.

***Redox activity of various Keggin-type POM catalysts***

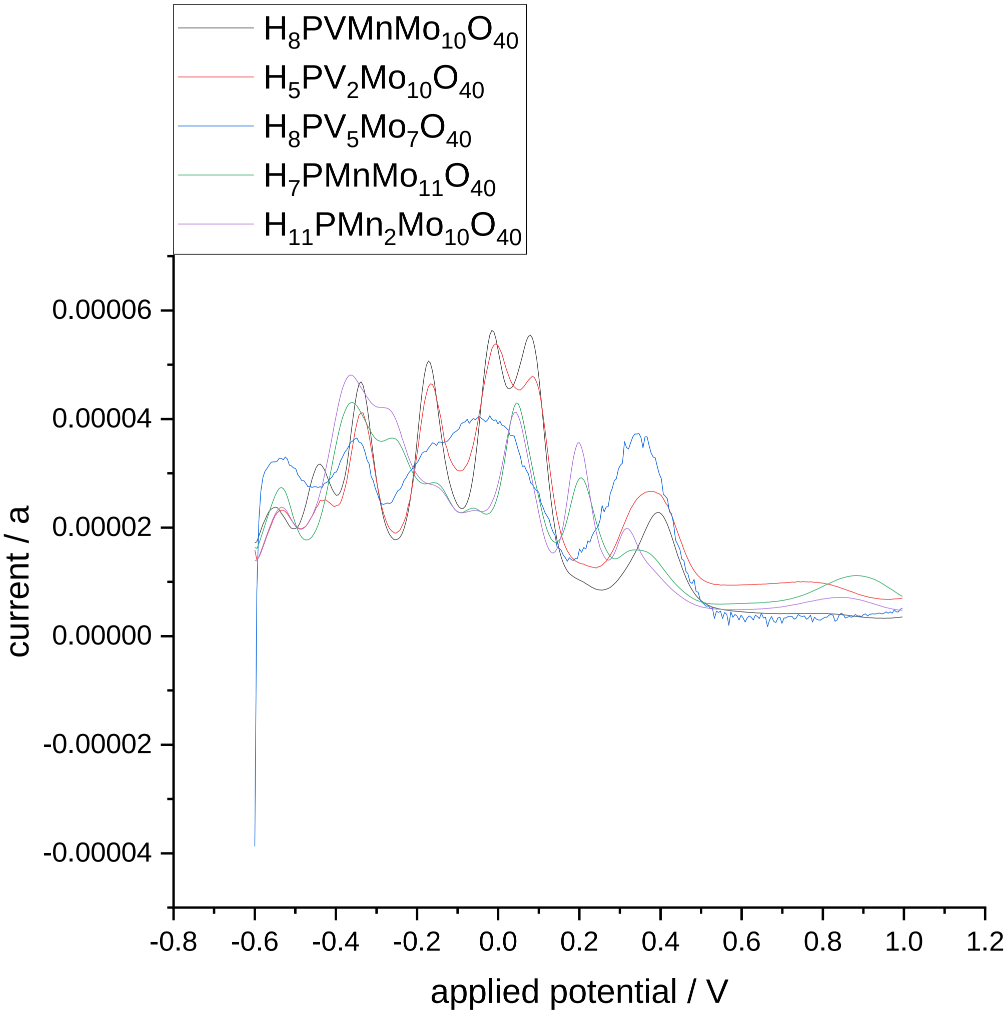


Figure S5: Square wave voltammetry data of the different catalysts in water (1 mmol $L^{-1}$).[1,2]

***Stability of TEA ([TEA][HSO$_4$]/MeOH 30 wt.%/70 wt.%.) with different catalysts***

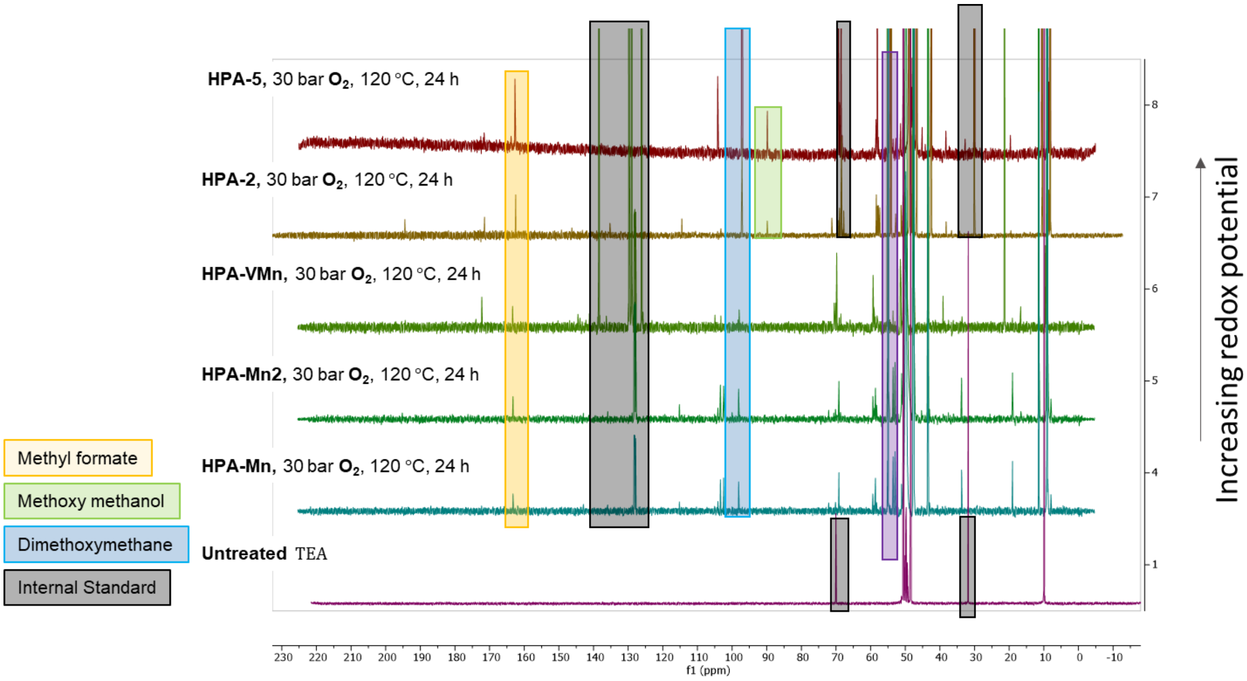


Figure S6: $^{13}$C NMR spectra of untreated TEA and with HPA-5, HPA-2, HPA-VMn, HPA-Mn2, and HPA-Mn under reaction conditions treated TEA. TEA is an abbreviation for the solvent mixture of [TEA][HSO4]/MeOH 30 wt.%/70 wt.%. The identified methanol oxidation products (methyl formate (163 ppm), methoxy methanol (91 ppm, 56 ppm) and dimethoxymethane (98 ppm, 51 ppm) and the internal standard (tert-butanol and acetonitrile) are marked.

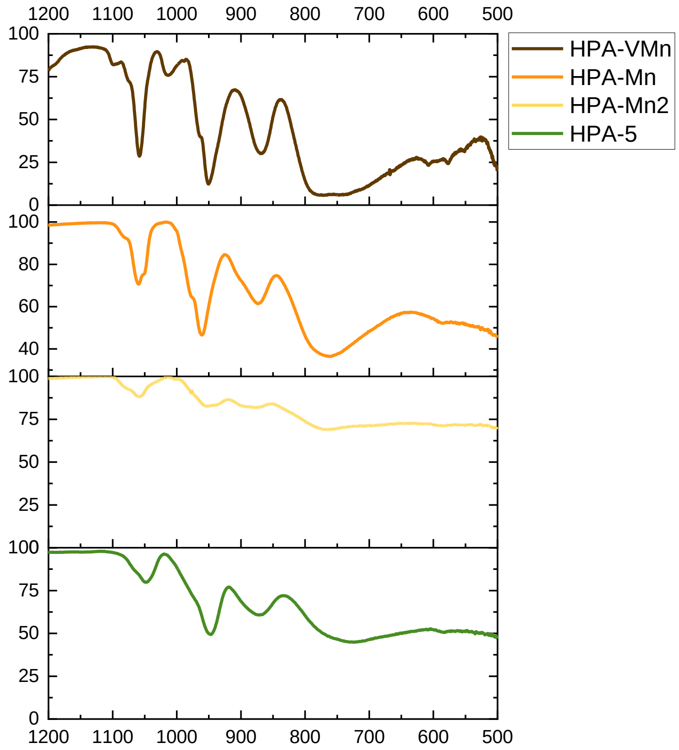


Figure S7: FT-IR spectra of different catalysts in TEA.

### ***Stability of DMBA ([DMBA][HSO$_4$]/MeOH 30 wt.%/70 wt.%.) with different catalysts***

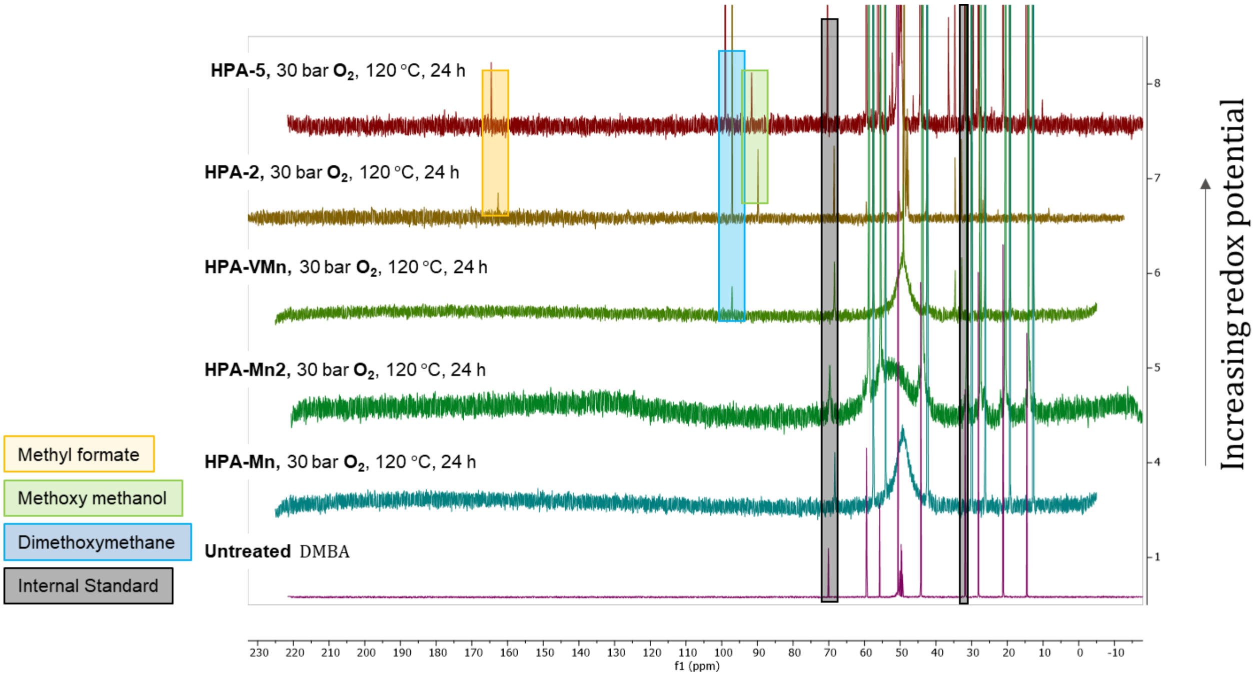


Figure S8: $^{13}$C NMR spectra of untreated DMBA and with HPA-5, HPA-2, HPA-VMn, HPA-Mn2, and HPA-Mn under reaction conditions treated DMBA. DMBA is an abbreviation for the solvent mixture of [DMBA][HSO4]/MeOH 30 wt.%/70 wt.%. The identified methanol oxidation products (methyl formate (163 ppm), methoxy methanol (91 ppm, 56 ppm) and dimethoxymethane (98 ppm, 51 ppm) and the internal standard (tert-butanol and acetonitrile) are marked.

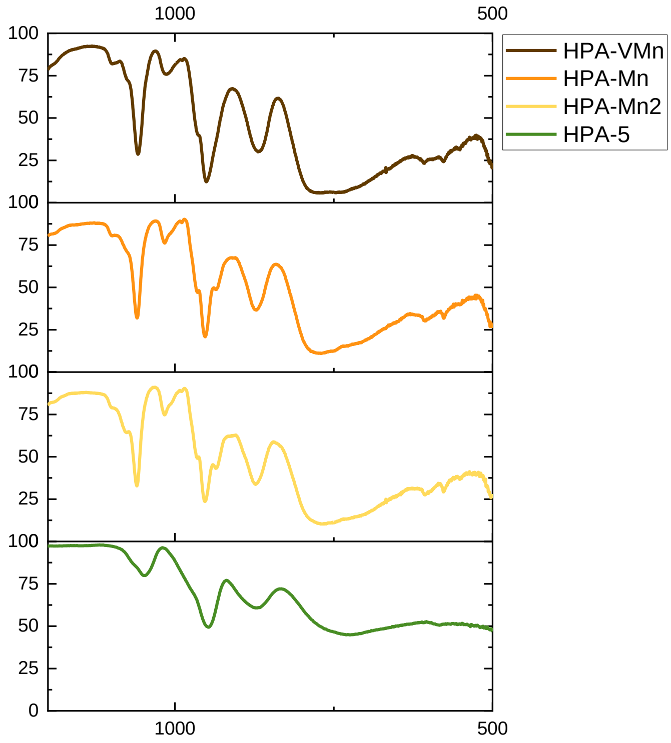


Figure S9: FT-IR spectra of different catalysts in DMBA.

***Stability of TBMP ([TBMP][MeSO$_4$]/MeOH 30 wt.%/70 wt.%.) with different catalysts***

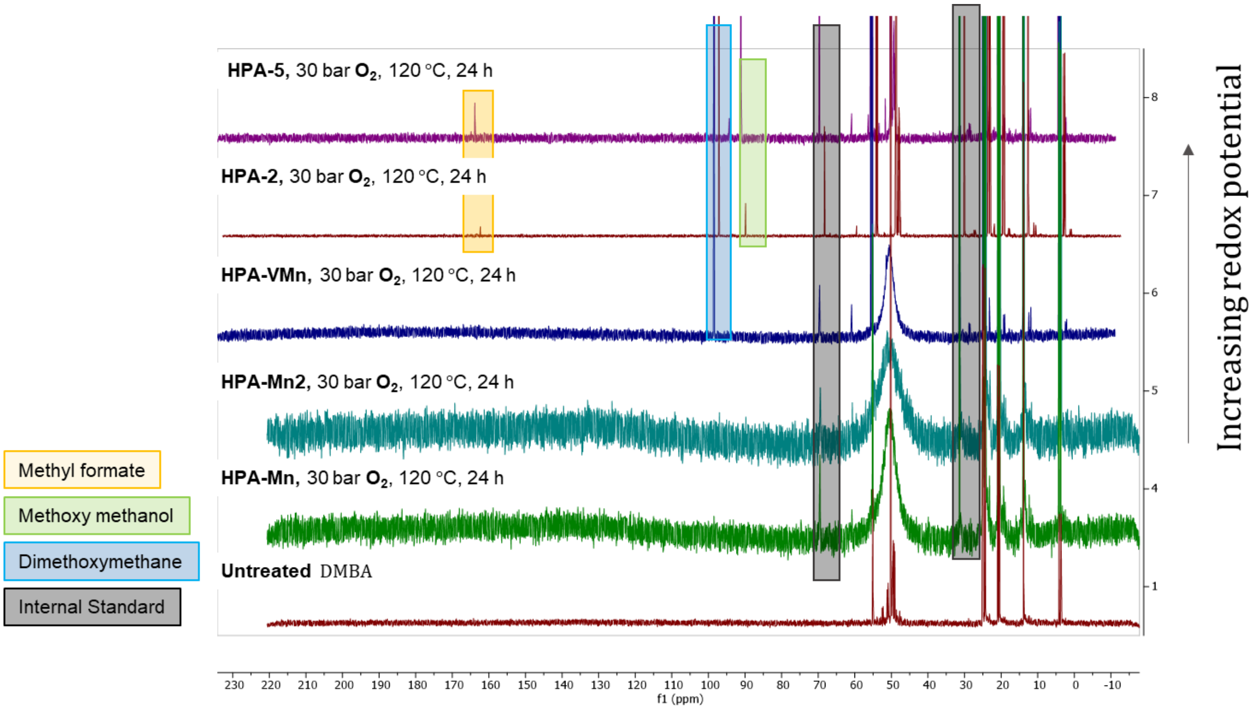


Figure S10: $^{13}$C NMR spectra of untreated TBMP and with HPA-5, HPA-2, HPA-VMn, HPA-Mn2, and HPA-Mn under reaction conditions treated TBMP. TBMP is an abbreviation for the solvent mixture of [TBMP][MeSO$_4$]/MeOH 30 wt.%/70 wt.%. The identified methanol oxidation products (methyl formate (163 ppm), methoxy methanol (91 ppm, 56 ppm) and dimethoxymethane (98 ppm, 51 ppm) and the internal standard (tert-butanol and acetonitrile) are marked.

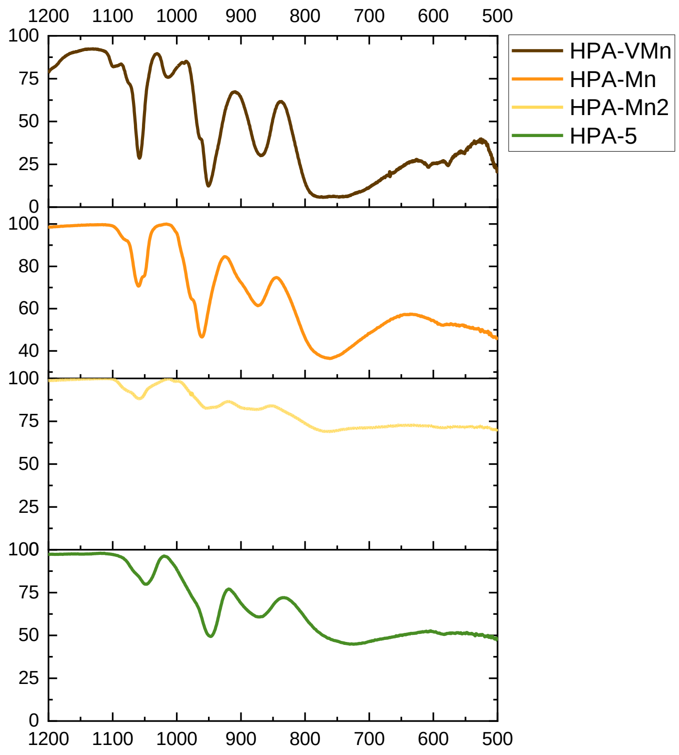


Figure S11: FT-IR spectra of different catalysts in TBMP.

**Investigating catalytic behavior under reaction conditions**

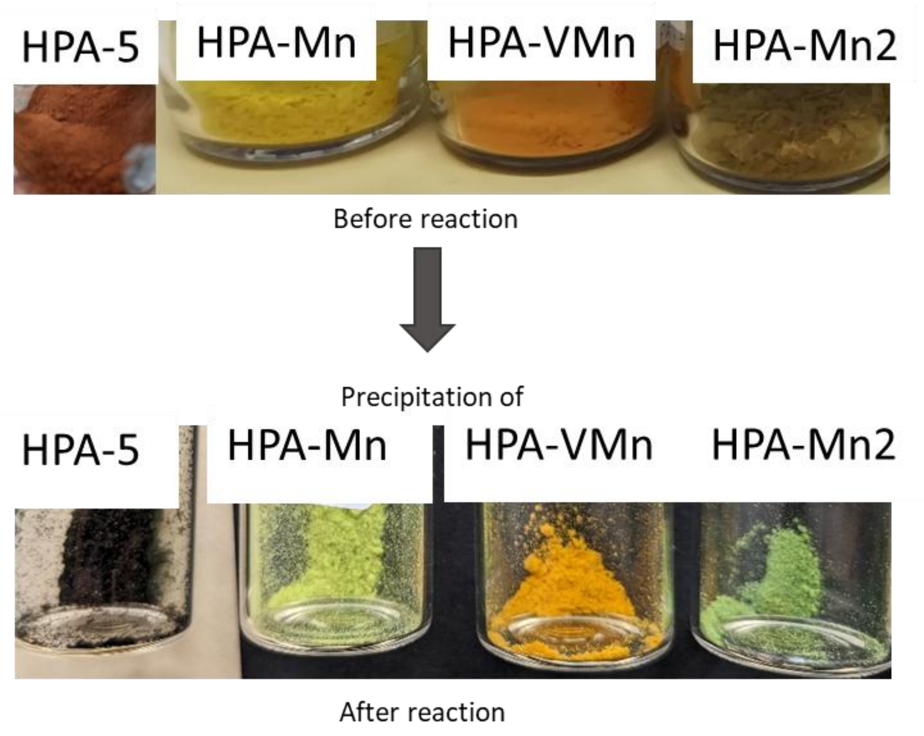


Figure S12: Catalysts before the reaction and the respective inorganic precipitates after the reaction.

Table S1: ICP-OES data as comparison between HPA-VMn as catalyst and the inorganic precipitate after the reaction with HPA-VMn and the methanolic TBMP solvent. *Reaction conditions: 120 °C, 24 h, 1000 rpm, 30 bar of oxygen at reaction temperature, 10 g solvent [TBMP][MeSO*$_4$*]/MeOH 30 wt.%/70 wt.% (methanolic TBMP solvent), 0.180 g HPA-VMn catalyst (1 mmol/g catalyst).*

| | | Mn | Mo | V | P |
|---|---|---|---|---|---|
| | Stoichiometry ideally for $H_8PVMnMo_{10}O_{40}$ | 1 | 10 | 1 | 1 |
| HPA-VMn ($H_8PVMnMo_{10}O_{40}$) | measurement [g/kg] | 30.1 | 501 | 27.5 | 20.3 |
| | stoichiometry | 1.05 | 10 | 1.03 | 1.25 |
| Inorganic precipitate before reaction | measurement [g/kg] | <0.05 | 41 | 1.8 | 5.5 |
| | stoichiometry | <0.02 | 10 | 0.82 | 4.16 |
| Inorganic precipitate after reaction | measurement [g/kg] | <0.05 | 38.94 | 1.14 | 5.96 |
| | stoichiometry | <0.02 | 10 | 0.55 | 4.74 |

**Detailed information about the crystal structure**

Table S2: Crystal data and structure refinement for CCDC 2422752.

| Crystal data and structure refinement for CCDC 2422752 | |
|---|---|
| Identification code | exp_270_autored |
| Empirical formula | $C_{39}Mo_{12}O_{40}P_4$ |
| Molecular weight/ g/mol | 2383.55 |
| Temperature/K | 120.00(11) |
| Crystal system | monoclinic |
| Space group | $P2_1/c$ |
| a/Å | 23.7676(2) |
| b/Å | 43.1368(5) |
| c/Å | 21.8129(2) |
| α/° | 90 |
| β/° | 91.5590(10) |
| γ/° | 90 |
| Volume/$Å^3$ | 22355.6(4) |
| Z | 12 |
| $\rho_{calc}g/cm^3$ | 2.125 |
| $\mu/mm^{-1}$ | 17.520 |
| F(000) | 13164.0 |
| Crystal size/$mm^3$ | 0.17 × 0.16 × 0.03 |
| Radiation | Cu Kα (λ = 1.54184) |
| 2Θ range for data collection/° | 4.54 to 161.106 |
| Index ranges | -29 ≤ h ≤ 28, -54 ≤ k ≤ 54, -22 ≤ l ≤ 27 |
| Reflections collected | 149069 |
| Independent reflections | 46582 [$R_{int}$ = 0.0548, $R_{sigma}$ = 0.0511] |
| Data/restraints/parameters | 46582/1417/2683 |
| Goodness-of-fit on $F^2$ | 1.056 |
| Final R indexes [I>=2σ (I)] | $R_1$ = 0.0725, $wR_2$ = 0.2018 |
| Final R indexes [all data] | $R_1$ = 0.0968, $wR_2$ = 0.2246 |
| Largest diff. peak/hole / e $Å^{-3}$ | 1.91/-1.78 |

Table S3: Selected bond lengths of the IL-POM salt obtained from the single crystal structure data.

| Bond | P-$O_p$ / Å | $O_P$-M / Å | M-$O_T$ / Å | M-$O_b$ / Å |
|---|---|---|---|---|
| This work | 1.531-1.540 | 2.424-2.431 | 1.674 | 1.884, 1.946 |
| $H_8[PVMnMo_{10}O_{40}]$[1] | 1.537 | 2.422 | 1.913 | 1.663 |
| $H_4[PVMo_{11}O_{40}]$[6] | 1.536 | 2.442 | 1.640 | 1.873 |

## X-ray photoelectron spectroscopy (XPS)

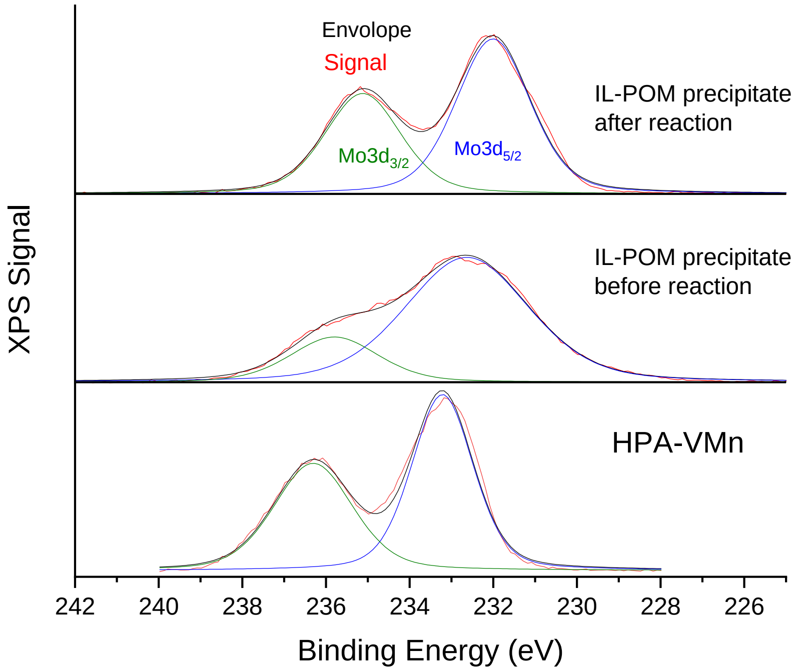


Figure S13: Comparison of the XPS signals of the Mo 3d bands of the inorganic precipitate (IL-POM precipitate) before and after the reaction and the initial HPA-VMn POM catalyst. *Reaction conditions: 120 °C, 24 h, 1000 rpm, 30 bar of oxygen at reaction temperature, 10 g solvent [TBMP][MeSO$_4$]/MeOH 30 wt.%/70 wt.% (methanolic TBMP system), 0.180 g catalyst HPA-VMn (1 mmol/g catalyst).*

Table S4: Relative surface concentrations of Mo, V, Mn and distribution of oxidation states of V and Mn determined via XPS.

| Sample | Mo | V | $V^{3+}$ | $V^{4+}$ | $V^{5+}$ | Mn | $Mn^{2+}$ | $Mn^{3+}$ | $Mn^{4+}$ |
|---|---|---|---|---|---|---|---|---|---|
| HPA-VMn | 9 | 0.50 | 0 | 0 | 100 | 0.50 | 0 | 25.7 | 74.7 |
| IL-POM precipitate before reaction | 9 | 0.98 | 0 | 16.7 | 83.3 | 0.32 | 1.7 | 57.7 | 40.7 |
| IL-POM precipitate after reaction | 9 | 0.37 | 14.1 | 45.6 | 40.4 | 0.00 | - | - | - |

## Characterization of the inorganic precipitate

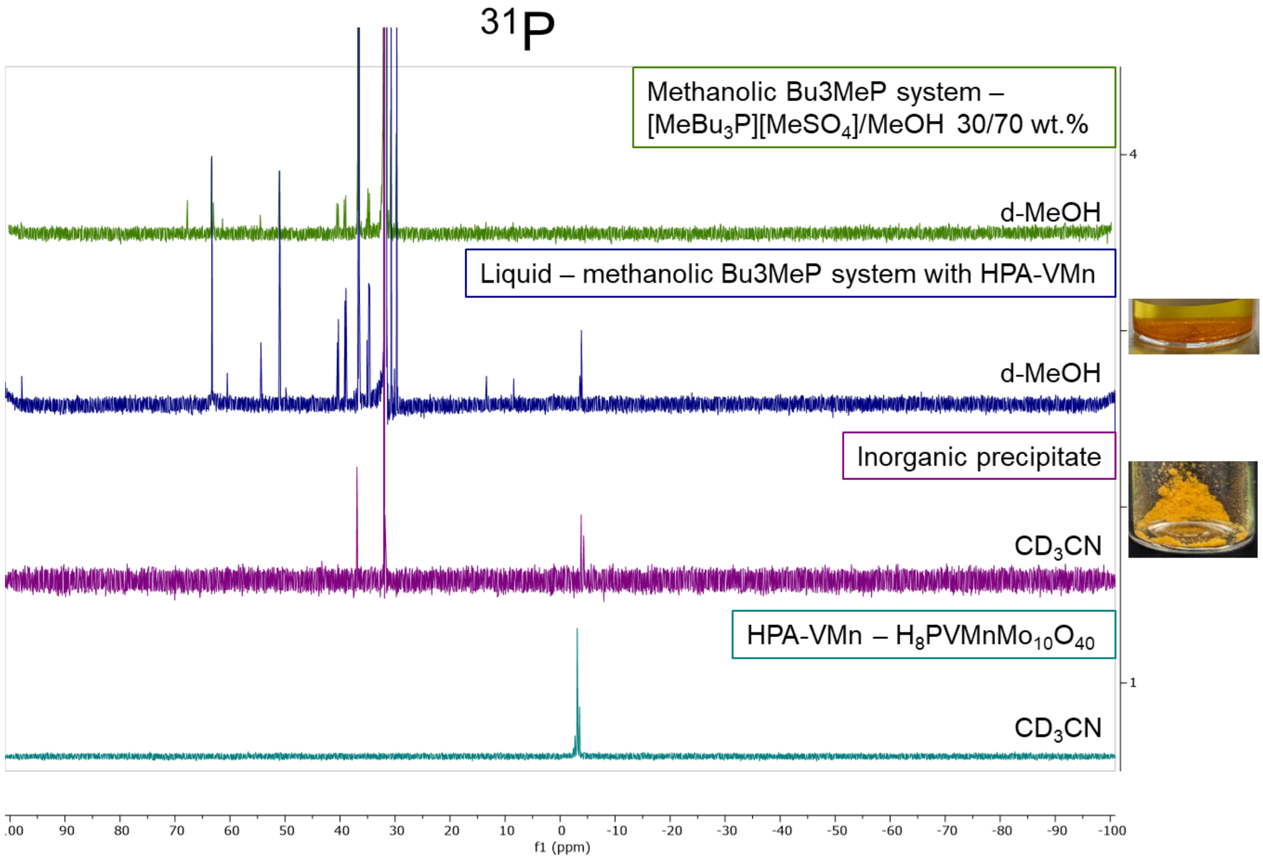


Figure S14: $^{31}$P NMR spectra of inorganic precipitate from TBMP and HPA-VMn (violet) diluted in deuterated acetonitrile, the liquid methanolic TBMP solution with dissolved HPA-VMn (blue) with deuterated methanol and HPA-VMn diluted in deuterated acetonitrile (turquoise) before reaction. In the $^{31}$P NMR spectra the initial methanolic TBMP system is displayed in green as a comparison to the other NMR spectra.

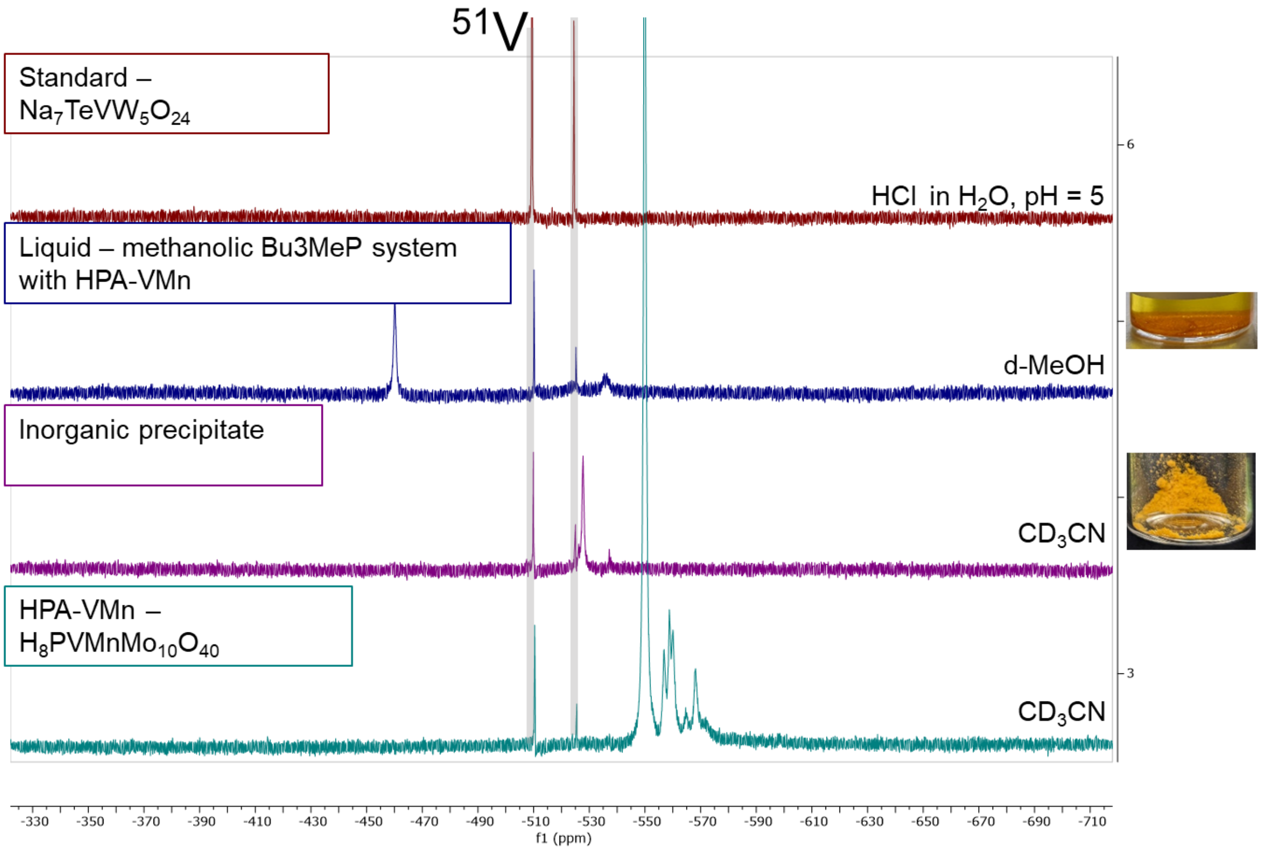


Figure S15: $^{51}V$ NMR spectra of inorganic precipitate from TBMP and HPA-VMn (violet) diluted in deuterated acetonitrile, the liquid methanolic TBMP solution with dissolved HPA-VMn (blue) with deuterated methanol and HPA-VMn diluted in deuterated acetonitrile (turquoise) before reaction. For a comparison an internal Standard of $Na_7TeVW_5O_{24}$ in deuterated water at pH = 5 was used (red) and marked in grey in the other spectra.

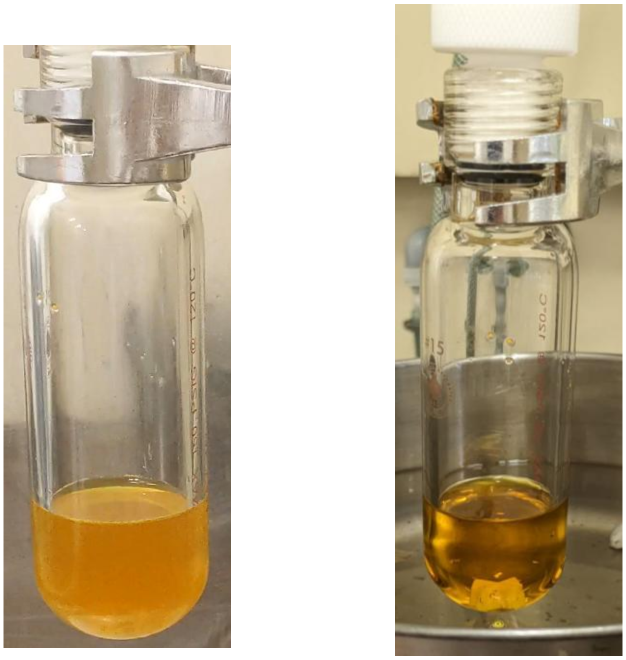

22 °C 95 °C

Figure S16: Comparison of pictures at 22 °C and 95 °C of the methanolic TBMP system with HPA-VMn as catalyst. The left picture shows an heterogeneous system with a solid inorganic precipitate and the right picture a homogeneous system where the precipitate is dissolved.

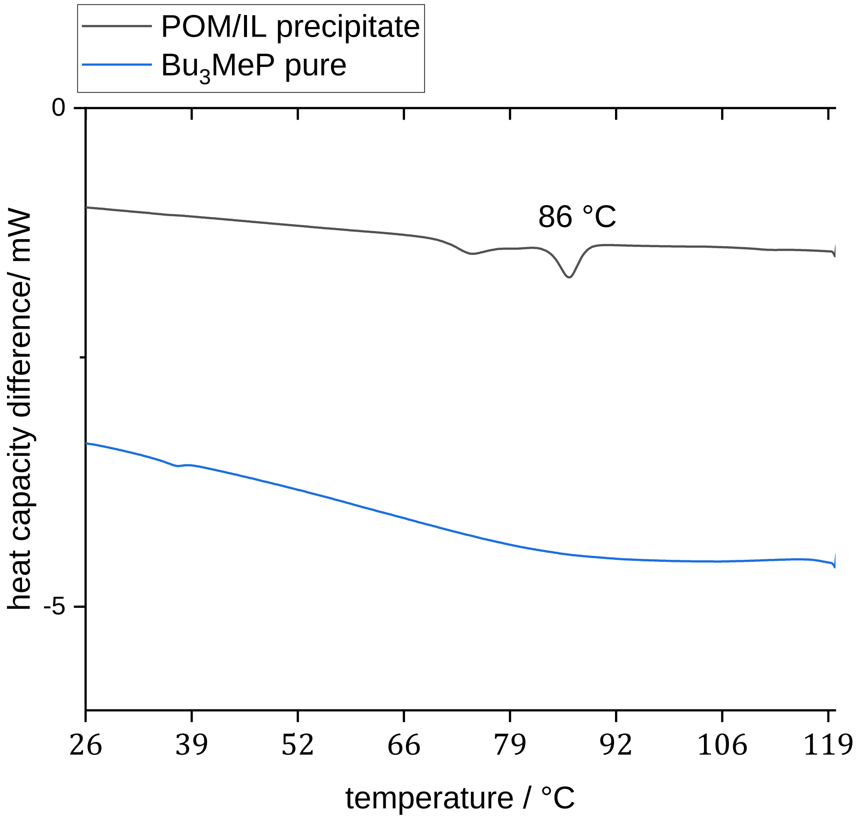


Figure S17: Comparison of DSC measurements of the POM/IL precipitate and the pure IL TBMP.

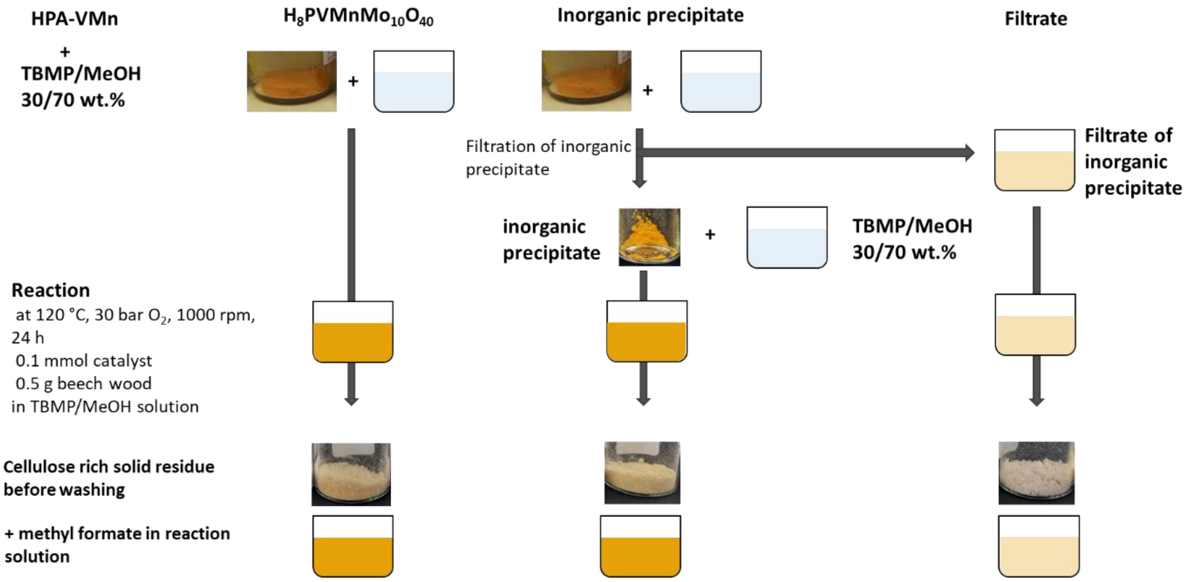


Figure S18: Schematic experimental flow sheet of work-up procedure.

## Time dependent optimization study

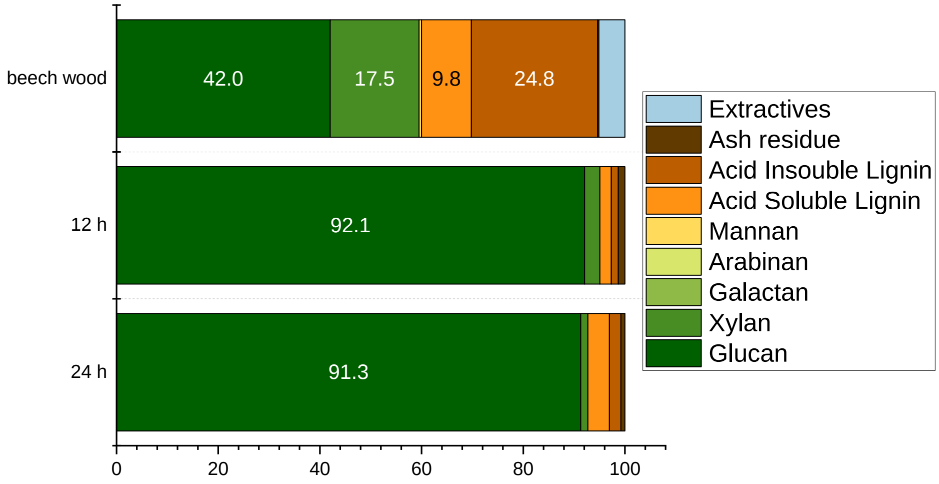


Figure S19: Compositional analysis via NREL of beech wood compared to cellulose-enriched solid residue after 12 h and 24 h of reaction time.

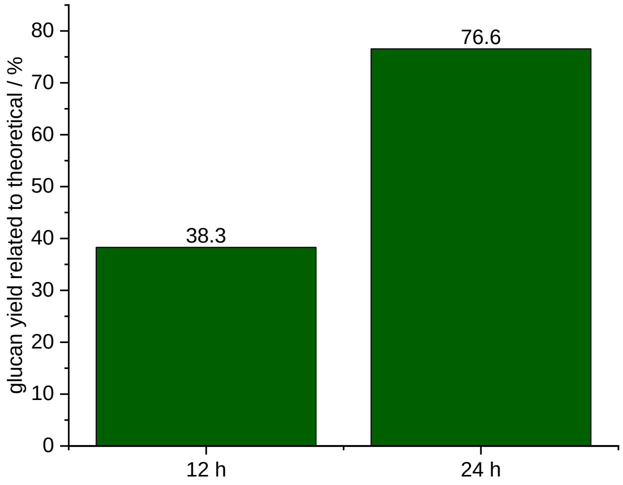


Figure S20: Enzymatic saccharification of the cellulose-rich solid after 12 h and 24 h of reaction time.

## Separation and purification of reaction products

### a) Processed solids

Table S5: Elemental content of V, Mo, P, S on solid after different washing procedures.

| Washing agent/ element content found on solid | Vanadium / wt.% determined via ICP-OES | Molybdenum / wt.% determined via ICP-OES | Phosphorous / wt.% determined via ICP-OES | Sulphur / wt.% determined via CHNS |
|---|---|---|---|---|
| MeOH | 0.24 | 14.41 | 1.69 | 0.27 |
| MeOH + acetone | 0.01 | 0.06 | 0.05 | 0.23 |
| MeOH + DMSO | <0.03 | <0.03 | <0.03 | 30.43 |

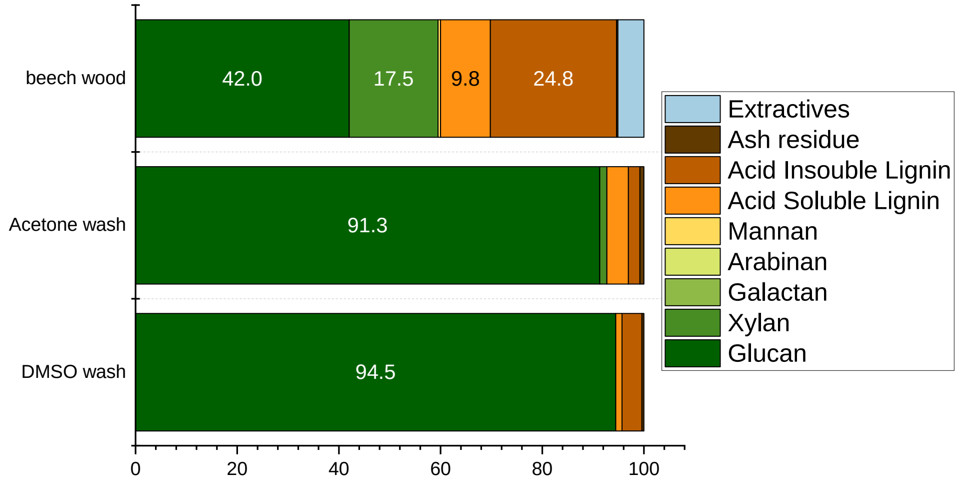


Figure S21: Comparison of compositional analysis of pure beech wood and the different washing agents acetone and DMSO.

**b) Liquid reaction product**

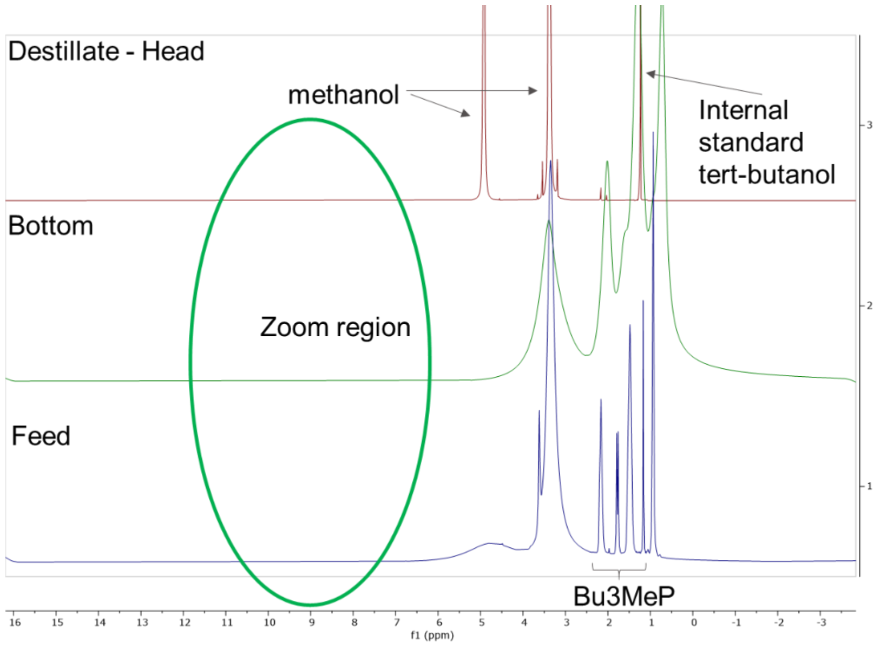


Figure S22: Comparison of the feed, the bottom of the distillation and the head in the $^{1}$HNMR spectrum.

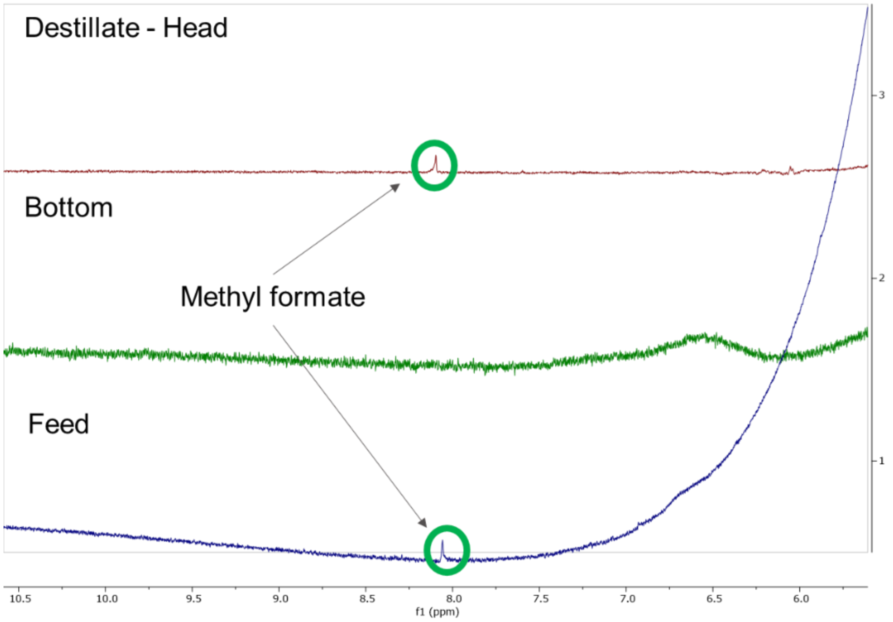


Figure S23: Zoom region of the comparison of the feed, the bottom of the distillation and the head in the $^{1}$H NMR spectrum.

## Substrate Screening

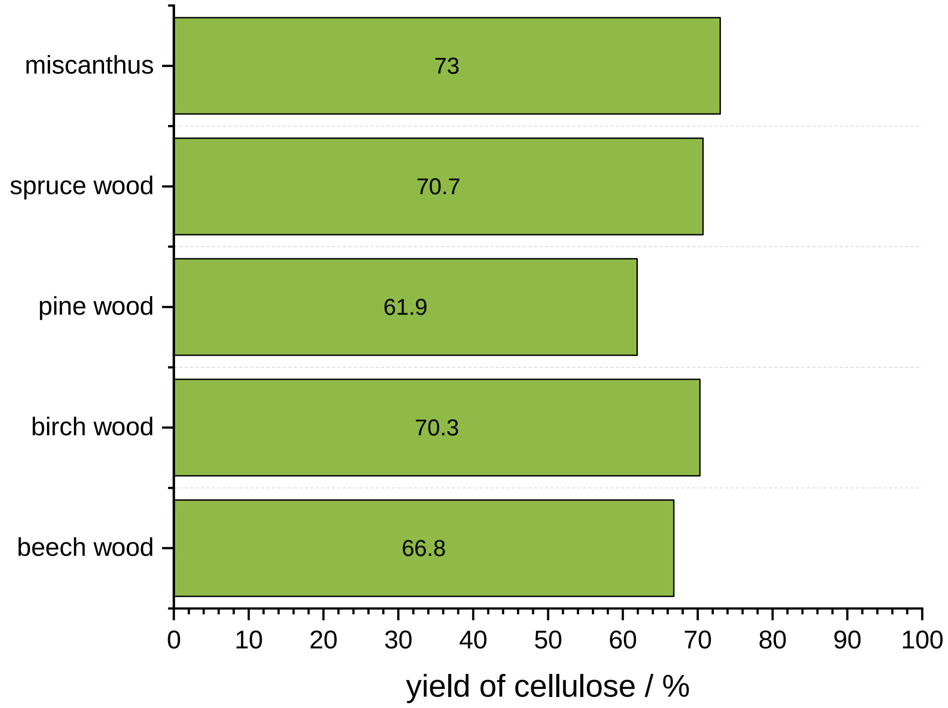


Figure S24: Yield of cellulose which could be extracted to the cellulose-enriched pulp from the initial substrate.

Table S6: All controlled experiments for the substrate screening in TBMP with HPA-VMn.

| | yield MF /% | error | yield $CO_2$ / % | error | yield CO / % | error | conversion in terms of mass / % | error |
|---|---|---|---|---|---|---|---|---|
| **Pine wood** | | | | | | | | |
| 1 | 15.01 | | 3.83 | | 0.46 | | 71.56 | |
| 2 | 14.57 | | 6.5 | | 1.08 | | 70.6 | |
| 3 | 17.9 | | 5.06 | | 0.83 | | 68.8 | |
| **average** | **15.83** | **1.48** | **5.13** | **1.09** | **0.79** | **0.25** | **70.32** | **1.14** |
| **spruce wood** | | | | | | | | |
| 1 | 15.47 | | 4.66 | | 0.79 | | 66.03 | |
| 2 | 5.89 | | 6.81 | | 0.93 | | 69.19 | |
| 3 | 8.67 | | 4.93 | | 0.7 | | 68.96 | |
| **average** | **10.01** | **4.02** | **5.47** | **0.96** | **0.81** | **0.09** | **68.06** | **1.44** |
| **Birch wood** | | | | | | | | |
| 1 | 16.29 | | 4.27 | | 0.66 | | 70.32 | |
| 2 | 17.08 | | 6.45 | | 0.95 | | 75.02 | |
| 3 | 18.98 | | 5.74 | | 0.88 | | 73.98 | |
| **average** | **17.45** | **1.13** | **5.49** | **0.91** | **0.83** | **0.12** | **73.11** | **2.02** |
| **Miscanthus** | | | | | | | | |
| 1 | 23.06 | | 5.38 | | 0.77 | | 61.44 | |
| 2 | 17.23 | | 3.06 | | 0.43 | | 67.8 | |
| 3 | 16.27 | | 5.26 | | 0.84 | | 67.4 | |
| **average** | **18.85** | **3.00** | **4.57** | **1.07** | **0.68** | **0.18** | **65.55** | **2.91** |
| **beech wood** | | | | | | | | |
| 1 | 21.7 | | 4.36 | | 0.8 | | 72.1 | |
| 2 | 15.34 | | 4.08 | | 0.91 | | 73.2 | |
| 3 | 16.05 | | 5.31 | | 0.83 | | 69.65 | |
| 4 | 16.98 | | 4.76 | | 0.99 | | 60.98 | |
| 5 | 21.46 | | 5.51 | | 0.95 | | 59.02 | |
| 6 | 22.25 | | 4.61 | | 1.34 | | 58.07 | |
| 7 | 19.97 | | 3.43 | | 0.84 | | 75.07 | |
| 8 | 20.23 | | 3.32 | | 0.84 | | 73.3 | |
| 9 | 19.83 | | 3.44 | | 0.79 | | 73.88 | |
| 10 | 21.9 | | 3.09 | | 0.72 | | 74.00 | |
| **average** | **19.83** | **2.44** | **4.19** | **0.82** | **0.91** | **0.16** | **69.27** | **6.24** |

Table S7: Control experiments for TBMP with beech wood.

| | conversion in terms of mass/ % | yield MF/ % | yield $CO_2$ /% | yield CO/ % |
|---|---|---|---|---|
| **Without catalyst** | | | | |
| 1 | 69% | 0.0% | 1% | 0.00% |
| 2 | 64% | 4.3% | 2% | 0.52% |
| 3 | 65.63% | 3.0% | 2.00% | 0.53% |
| 4 | 66.22% | 6.9% | 2.12% | 0.49% |
| 5 | 66.23% | 2.1% | 1.70% | 0.48% |
| 6 | 64.14% | 2.7% | 2.30% | 0.54% |
| 7 | 65.16% | 2.03% | 1.34% | 0.36% |
| 8 | 66.12% | 1.88% | 1.13% | 0.39% |
| 9 | 67.16% | 2.19% | 1.13% | 0.38% |
| **average** | **65.9%** | **2.8%** | **1.6%** | **0.4%** |
| **error** | **1.3%** | **1.8%** | **0.4%** | **0.2%** |
| **HPA-5** | | | | |
| 1 | 69.0% | 13.9% | 3.60% | 0.85% |
| 2 | 69.80% | | | |
| 3 | 68.60% | 3.6% | 3.84% | 0.64% |
| 4 | 69.10% | 10.2% | 6.89% | 1.08% |
| 5 | 70.30% | 2.8% | 6.51% | 1.03% |
| 6 | 67.50% | 5.5% | 2.49% | 0.45% |
| 7 | 66.90% | 9.1% | 3.67% | 0.92% |
| 8 | 69.90% | 2.1% | 3.96% | 0.59% |
| 9 | 70.50% | 3.7% | 3.75% | 0.57% |
| 10 | | | | |
| **average** | **69.1%** | **6.3%** | **4.3%** | **0.8%** |
| **error** | **1.1%** | **3.9%** | **1.6%** | **0.2%** |

Table S8: Control experiments for DMBA with beech wood.

| HPA-5 | conversion in terms of mass/ % | yield MF/ % | yield $CO_2$ /% | yield CO/ % |
|---|---|---|---|---|
| 1 | 58% | 2.9% | 2.86% | 0.59% |
| 2 | 63% | 1.8% | 2.94% | 0.54% |
| 3 | 72.2% | 6.1% | 4.28% | 0.93% |
| **Average** | **64.4%** | **2.7%** | **2.6%** | **0.7%** |
| **Error** | **5.8%** | **2.2%** | **1.5%** | **0.2%** |
| **Without catalyst** | | | | |
| 1 | 71% | 4.3% | 2.83% | 0.0091 |
| 2 | 62 % | 5.1% | 4.91% | 1.32% |
| 3 | No data (n.d) | 4.8% | 4.69% | 1.25% |
| 4 | n.d | 10.6% | 3.21% | 1.04% |
| 5 | n.d | 5.0% | 2.77% | 0.86% |
| **Average** | **66.3%** | **5.9%** | **3.7%** | **1.1%** |
| **Error** | **4.2%** | **2.3%** | **0.9%** | **0.2%** |

Table S9: Control experiments for TEA with beech wood.

| Without catalyst | conversion in terms of mass/ % | yield MF/ % | yield $CO_2$ /% | yield CO/ % |
|---|---|---|---|---|
| 1 | 46.26% | 0.55% | 0.01% | 0.00% |
| 2 | 50% | 0.1% | 3.50% | 0.00% |
| 3 | 35% | 0.2% | 1.65% | 0.00% |
| 4 | 73.70% | 0.0% | 0.54% | 0.00% |
| 5 | 44.80% | 0.1% | n.d. | 0.00% |
| **Average** | **49.87%** | **0.18%** | **1.32%** | **0.00%** |
| **Error** | **12.85%** | **0.19%** | **1.46%** | **0.00%** |
| **Without catalyst** | | | | |
| 1 | 33.60% | 0.16% | 0.57% | 0% |
| 2 | 24% | 0.2% | 1% | 0.00% |
| 3 | 16.20% | 0.18% | 0.42% | 0.14% |
| 4 | 15.80% | 0.23% | 0.44% | 0.12% |
| 5 | 17.95% | 0.3% | 0.44% | 0.13% |
| 6 | 18.98% | 0.3% | 0.37% | 0.13% |
| 7 | 19.00% | 0.2% | 0.37% | 0.13% |
| 8 | 17.60% | 0.27% | 0.41% | 0.15% |
| 9 | 17.64% | 0.28% | 0.38% | 0.14% |
| 10 | 16.88% | 0.33% | 0.42% | 0.13% |
| **Average** | **19.80%** | **0.19%** | **0.52%** | **0.11%** |
| **Error** | **5.13%** | **0.41%** | **0.29%** | **0.05%** |

## References


1 J.-C. Raabe, J. Albert and M. J. Poller, *Chemistry (Weinheim an der Bergstrasse, Germany)*, 2022, **28**, e202201084.

2 J.-C. Raabe, M. J. Poller, D. Voß and J. Albert, *ChemSusChem*, 2023, **16**, e202300072.

3 O. V. Dolomanov, L. J. Bourhis, R. J. Gildea, J. A. K. Howard and H. Puschmann, *J Appl Crystallogr*, 2009, **42**, 339–341.

4 G. M. Sheldrick, *Acta crystallographica. Section A, Foundations and advances*, 2015, **71**, 3–8.

5 G. M. Sheldrick, *Acta crystallographica. Section C, Structural chemistry*, 2015, **71**, 3–8.

6 T. Esser, A. Wassenberg, J.-C. Raabe, D. Voß and J. Albert, *ACS Sustainable Chem. Eng.*, 2024, **12**, 543–560.
